# Identification of m^5^C-related lncRNAs signature to predict prognosis and therapeutic responses in esophageal squamous cell carcinoma patients

**DOI:** 10.1038/s41598-023-41495-6

**Published:** 2023-09-04

**Authors:** Yuan Ma, Yuchen Sun, Xu Zhao, Jing Li, Xing Fu, Tuotuo Gong, Xiaozhi Zhang

**Affiliations:** Department of Radiation Oncology, The First Affiliated Hospital of Xi’an Jiaotong University, Yanta West Road 277, Xi’an, 710061 Shaanxi China

**Keywords:** Cancer models, Gastrointestinal cancer, Tumour biomarkers

## Abstract

Esophageal squamous cell carcinoma (ESCC) has a dismal prognosis because of atypical early symptoms and heterogeneous therapeutic responses. 5-methylcytosine (m^5^C) modification plays an important role in the onset and development of many tumors and is widespread in long non-coding RNA (lncRNA) transcripts. However, the functions of m^5^C and lncRNAs in ESCC have not been completely elucidated. Herein, this study aimed to explore the role of m^5^C-related lncRNAs in ESCC. The RNA-seq transcriptome profiles and clinical information were downloaded from the TCGA-ESCC database. Pearson analysis was used to identify m^5^C-related lncRNAs. Then we established the m^5^C-related lncRNAs prognostic signature (m^5^C-LPS) using univariate Cox and least absolute shrinkage and selection operator (LASSO) regression analysis. Then, the prognostic value of m^5^C-LPS was evaluated internally and externally using the TCGA-ESCC and GSE53622 databases through multiple methods. We also detected the expression of these lncRNAs in ESCC cell lines and patient tissues. Fluorescence in situ hybridization (FISH) was used to detect the prognostic value of specific lncRNA. In addition, clinical parameters, immune status, genomic variants, oncogenic pathways, enrichment pathways, and therapeutic response features associated with m^5^C-LPS were explored using bioinformatics methods. We constructed and validated a prognostic signature based on 9 m^5^C-related lncRNAs (*AC002091.2*, *AC009275.1*, *CAHM*, *LINC02057.1*, *AC0006329.1*, *AC037459.3*, *AC064807.1*, *ATP2B1-AS1*, and *UBAC2-AS1*). The quantitative real-time polymerase chain reaction (qRT-PCR) revealed that most lncRNAs were upregulated in ESCC cell lines and patient tissues. And *AC002091.2* was validated to have significant prognostic value in ESCC patients. A composite nomogram was generated to facilitate clinical practice by integrating this signature with the N stage. Besides, patients in the low-risk group were characterized by good clinical outcomes, favorable immune status, and low oncogenic alteration. Function enrichment analysis indicated that the risk score was associated with mRNA splicing, ncRNA processing, and DNA damage repair response. At the same time, we found significant differences in the responses to chemoradiotherapy between the two groups, proving the value of m^5^C-LPS in treatment decision-making in ESCC. This study established a novel prognostic signature based on 9 m^5^C-related lncRNAs, which is a promising biomarker for predicting clinical outcomes and therapeutic response in ESCC.

## Introduction

Esophageal cancer (EC) ranks eighth and sixth in terms of incidence and mortality worldwide, respectively^1^. Among primary esophageal cancers, approximately 88% are classified as esophageal squamous cell carcinoma (ESCC), which exhibits a relatively low 5-year survival rate ranging from 5 to 25%^2–5^. Currently, extensive research on molecular mechanisms has yielded promising precision cancer treatment strategies for numerous cancers^6^. Recently, growing research has highlighted the role of RNA post-transcriptional modifications 5-methylcytosine (m^5^C) on tumor development^7,8^. Several studies have provided evidence that m^5^C can influence the development of ESCC^9,10^. However, the precise impact of m^5^C on ESCC remains unclear and needs further investigation.

The reversible RNA post-transcription modification m^5^C, similar to N6-methyladenosine (m^6^A), has got enormous attention and can dynamically regulate RNA stability, translation, splicing, and exportation^7,8,11^. The m^5^C is a type of cytosine methylation that involves the addition of a methyl group to the fifth carbon position and is regulated by several enzymes including “writers” (methyltransferases: *NSUN1-7*, *DNMT1*, *DNMT2* also named *TRDMT1*, *DNMT3A*, and *DNMT3B*), “erasers” (demethylases: *TET1-3*), and “readers” (*YBX1* and *ALYREF*)^12–14^.

Mounting evidence suggests that dysregulated expression of long non-coding RNAs (lncRNAs) plays a critical role in tumor development and response to therapy^15–17^. For instance, lncRNA *CASC9* has been shown to promote ESCC metastasis^18^. While m^5^C was initially found in tRNA and rRNA, emerging evidence suggests that it is also widespread presence in mRNAs and non-coding RNAs^19–21^. And the methylation density around the transcriptional start site of lncRNAs is higher than that of protein-coding genes^21^. Upregulated *NSUN2-*mediated *NMR* methylation in ESCC, resulting in cancer metastasis and drug resistance^22^, which suggested that m^5^C-methylated lncRNAs can regulate the biological function of cancer. However, the evidence for m^5^C in regulating lncRNAs in ESCC is limited and requires further research.

In this study, we aimed to investigate the function of m^5^C-related lncRNAs in ESCC and construed m^5^C-related lncRNAs prognostic signature (m^5^C-LPS) based on the TCGA-ESCC cohort. Additionally, we also explored the relationship between the m^5^C-LPS and clinical prognostic, immune status, genomic variants, enrichment pathways, as well as drug sensitivity in ESCC.

## Methods

### Patients cohorts

We have included patients diagnosed with ESCC in The Cancer Genome Atlas (TCGA) program (https://portal.gdc.cancer.gov/repository?facetTab=cases). Patients without complete clinical information and transcriptome profiling, or diagnosed with esophageal adenocarcinomas were excluded. Finally, the transcriptome, clinicopathologic, and somatic mutation data of 80 ESCC and 11 adjacent normal tissues were downloaded. Additionally, RNA microarray profiles and corresponding clinical information of 60 ESCC patients were downloaded from the Gene Expression Omnibus database (GSE53622, https://www.ncbi.nlm.nih.gov/geo/query/acc.cgi?acc=gse53622). The clinicopathological parameters of TCGA-ESCC and GSE53622 cohorts were summarized in Table S1. Immunohistochemical staining images of normal esophageal tissues were obtained from the Human Protein Atlas (HPA) (https://www.proteinatlas.org/). And this study started in February 2022 and finished in July 2022.

Paraffin embedded sections of 54 ESCC patients were obtained from the First Affiliated Hospital of Xi’an JiaoTong University. Tissues were collected during surgery and were used for Fluorescence in situ hybridization (FISH) examination. And 14 ESCC and corresponding normal tissue samples were collected for the detection of lncRNA expression.

### Identification of regulators of m^5^C and co-expression lncRNAs

We identified 16 m^5^C regulators from previous literature and extracted their expression from RNA-seq profiles of ESCC and adjacent normal tissues. Then, the differential expression of 16 m^5^C regulators was determined in the ESCC tissues versus adjacent normal tissues. The differential expression of these regulators was analyzed, and their interrelationships were visualized using the ‘corrplot’ R package. A protein–protein interaction (PPI) network of m^5^C regulators was constructed using the STRING database (https://cn.string-db.org/) with the gene interaction score ≥ 0.5^23^. We selected the lncRNAs existed in both TCGA-ESCC and GSE53622 cohorts for widespread use of m^5^C-LPS. Pearson correlation coefficient was calculated between the expression of 16 m^5^C regulators and lncRNAs using the built-in function ‘cor.test’ in R. We identified 4279 m^5^C-related lncRNAs with |correlation coefficient| > 0.35 and the *p*-value < 0.01 for further analysis.

### Construction and validation of m^5^C-related lncRNA prognosis signature

We used the ‘survival’ R package to perform univariate Cox regression analysis on the candidate m^5^C-related lncRNAs, filtering out those with significant prognostic value (*p* < 0.05). Then, we used the least absolute shrinkage and selection operator (LASSO) regression analysis with the ‘glmnet’ R package to establish a prognostic signature and calculate the coefficients for each lncRNA^24–26^. These coefficients were used to generate a risk score formula: $\mathrm{Risk \,Score}=\sum_{i}coefficient\, of \,m5C\, related\, lncRNAi\times lncRNAi \,expression\, level$. Patients were stratified into high- and low-risk groups based on their calculated risk scores. Kaplan–Meier (K–M) analysis was performed to assess the overall survival (OS) of different groups, and time-dependent receiver operating characteristic (ROC) curve analysis was used to evaluate the predictive value of the risk score with the ‘survivalROC’ R package.

### Clinical relevance investigation

A Sankey diagram was used to illustrate the one-to-one match between the m^5^C genes, m^5^C-related lncRNAs, and the corresponding risk types. Furthermore, a correlation circle graph was generated using the ‘corrplot’ and ‘circlize’ R package to visualize the co-expression status of the 9 identified lncRNAs. We also investigated the association between m^5^C-LPS and clinicopathological parameters. Both univariate and multivariate Cox regression analyses were conducted to investigate the independent value of the m^5^C-LPS and other parameters. Based on the significant prognostic variables, we constructed a nomogram to predict 1-, 2-, and 3-year survival rates using the ‘rms’ R package. And calibration curves were used to verify the agreement between nomogram-predicted survival and actual survival probabilities. Additionally, we evaluated the prognostic value of clinicopathological features by using ROC curves and calculating the area under the curve (AUC).

### Evaluation of signaling pathways enrichment

We conducted functional enrichment analyses based on gene ontology (GO)^27^, Kyoto Encyclopedia of Genes and Genomes (KEGG)^28^, and Reactome^29^ databases to explore the biological functions and pathways associated with m^5^C-LPS through ‘clusterProfiler’^30^ and ‘ReactomePA’ R package.

### Estimation of the tumor microenvironment signatures

Estimate^31^, single-sample gene set enrichment analysis (ssGSEA)^32^, Cibersort^33^, and xCell^34^ algorithms were utilized to estimate the relative abundance of immune and stromal cells in the tumor microenvironment. We also calculated the Pearson coefficients between risk scores and immune checkpoint genes and immunomodulators, such as chemokines, receptors, MHC, immunoinhibitors, and immunostimulators, which were obtained from the TISIDB database^35^.

### Characterization of genetic alteration

The ‘maftools’ R package was utilized to identify the top 20 mutated genes based on the mutation rate across low- and high-risk groups^36^. Subsequently, we further investigated the fraction of affected samples and pathways based on alterations in 10 canonical oncogenic signaling pathways for different risk groups^37^.

### Drug sensitivity analysis

We employed the ‘pRRophetic’ R package^38^ to predict the half-maximal inhibitory concentration (IC50) for each patient using three publicly available drug sensitivity databases (Cancer Genome Project (CGP)^39^, Cancer Therapeutics Response Portal (CTRP)^40^, and Genomics of Drug Sensitivity in Cancer (GDSC)^41^). Additionally, we utilized the genomic-adjusted radiation dose (GARD) model^42^ to predict the radiotherapy response of each patient, with higher GARD values indicating increased sensitivity to radiotherapy.

### Cell lines and reagents

The human normal esophageal cell line HET-1A was purchased from American Type Culture Collection (ATCC, Virginia, USA), and the human ESCC cell lines TE-1 and KYSE150 were purchased from the Cell Bank of the Chinese Academy of Sciences Typical Culture Preservation Committee (Shanghai, China). HET-1A was cultured in Dulbecco’s Modified Eagle’s Medium (DMEM, Gibico, USA) supplemented with 10% fetal bovine serum (FBS, Gibco, USA), while TE-1, and KYSE150 were cultured in Roswell Park Memorial Institute (RPMI) 1640 medium (Gibico, USA) supplemented with 10% FBS. All cells were cultured in a 5% CO_2_ incubator at 37 °C.

### Total RNA extraction and real-time quantitative PCR

Total RNA was extracted using the RNAfast200 kit (Fastagen, China) according to the manufacturer’s instructions. RNA concentration was quantified using NanoDrop 3000 (ThermoFisher, USA). Then, 1.0 μg of total RNA in a 20 μl reaction system was reversely transcribed into cDNAs using Evo M-MLV RT Kit with gDNA Clean for polymerase chain reaction (PCR, Accurate Biotechnology, China). Quantitative real-time PCR (qRT–PCR) was performed using 2$\times$ RealStar Green Fast Mixture (GeneStar Technology, China). GAPDH expression was used as an internal reference. The relative expression level of lncRNAs was calculated using the 2^−ΔΔCT^ method. Each experiment was performed in triplicate. The primer sequences used in this study are listed in Table S2.

### FISH assay

The FISH probe of lncRNA *AC002091.2* was synthesized by Servicebio (Wuhan, China). Paraffin embedded sections were dewaxed, rehydrated, digested, and dehydrated with dimethylbenzene, graded ethanol, protease K. Then the FISH probe was added to the hybridization mixture and incubated overnight. Next, the section was washed in the dark with washing buffer containing saline sodium citrate and PBS. Sections were stained with DAPI for 10 min and then visualized by fluorescence microscope.

### Statistical analysis

All statistical analyses were performed using R software (version 4.1.1) and GraphPad Prism (version 8.0, USA). The differences between the two groups were compared using student’s t-test, while one-way analysis of variance (ANOVA) was used for multiple groups. Fisher’s exact test was used to compare categorical variables. The correlation between two continuous variables was analyzed using Pearson’s test. *p*-value < 0.05 was considered statistically significant.

### Ethics approval and consent to participate

This study was approved by the Ethics Committee of The First Affiliated Hospital of Xi’an Jiaotong University (Approval Number: 2017-146).

## Results

To facilitate the comprehension of the study, a schematic diagram is presented in Fig. S1.

### Expression patterns of m^5^C regulators in ESCC and normal esophageal tissue

**Figure 1:**
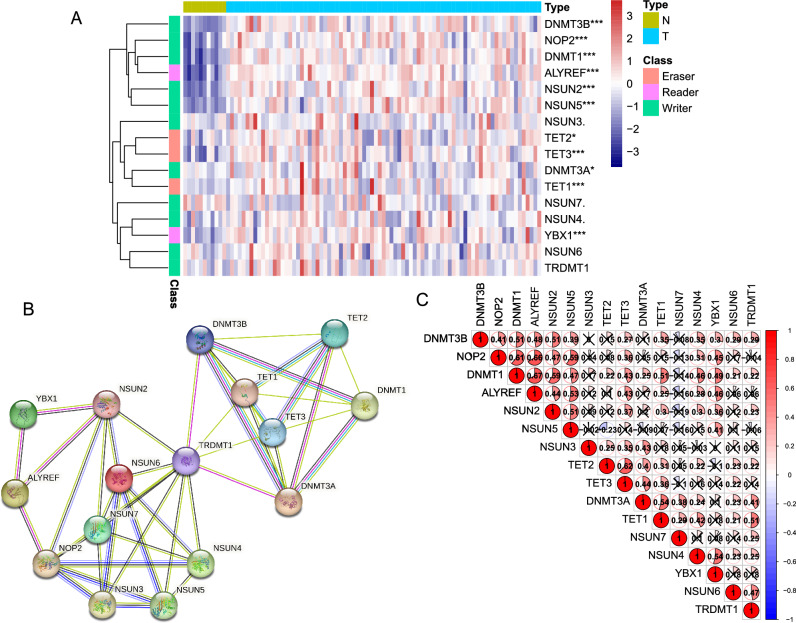
The expression pattern and interactive landscape of the m^5^C regulators in the TCGA-ESCC database. **(A)** Heatmap presenting the expression of 16 m^5^C regulators in normal esophageal (N) and ESCC (T) tissues from TCGA-ESCC database. *p* < 0.1, *p < 0.05, **p < 0.01, and ***p < 0.001. **(B)** Protein–protein interaction (PPI) network showing the interaction between m^5^C regulators. **(C)** Heatmap showing the Pearson correlation among 16 m^5^C regulators.

We extracted the expression profiles of 16 m^5^C regulators in the TCGA-ESCC cohort and subsequently compared their expression levels between 80 ESCC tumor samples and 11 normal adjacent samples. Our analysis revealed that the expression of most genes, including *DNMT3B, NOP2, DNMT1, ALYREF, NSUN2, NSUN5, TET2, TET3, DNMT3A, TET1,* and *YBX1* were significantly higher in ESCC tissues than in normal adjacent tissues (Fig. 1A). Moreover, the immunohistochemical staining images of normal esophageal tissues from HPA showed that 10 of 15 m^5^C regulators were not more than medium expression, while *NSUN3* and *NSUN5* were not detected (Fig. S2). To investigate the interrelationships among these 16 m^5^C regulators, we obtained a PPI network using the STRING database. After setting the minimum interaction score as 0.5, we identified the PPI network contains all m^5^C genes and 90 edges (Fig. 1B). And *TRDMT1* was found to be the hub gene of the network with 11 edges (Table S3). In addition, the correlation analysis revealed significant positive correlations between *TRDMT1* and other 8 m^5^C genes. Interestingly, all m^5^C regulators showed a general positive correlation, with *DNMT1* exhibiting the highest correlation with *ALYREF* (r = 0.67) (Fig. 1C).

### Construction of the m^5^C‑LPS in the TCGA database

Subsequently, we performed Pearson analysis based on the lncRNAs and m^5^C regulators in TCGA-ESCC profiles, and a total of 4279 lncRNAs were significantly correlated with m^5^C regulators (|Pearson coefficient| > 0.35 and *p* < 0.01). After filtering lncRNAs with the sum expression < 0.01, univariate Cox regression analysis was conducted to further explore the m^5^C-related lncRNAs associated with prognosis. Finally, we identified 41 lncRNAs that were significantly associated with the OS of ESCC patients (Table S4).

**Figure 2:**
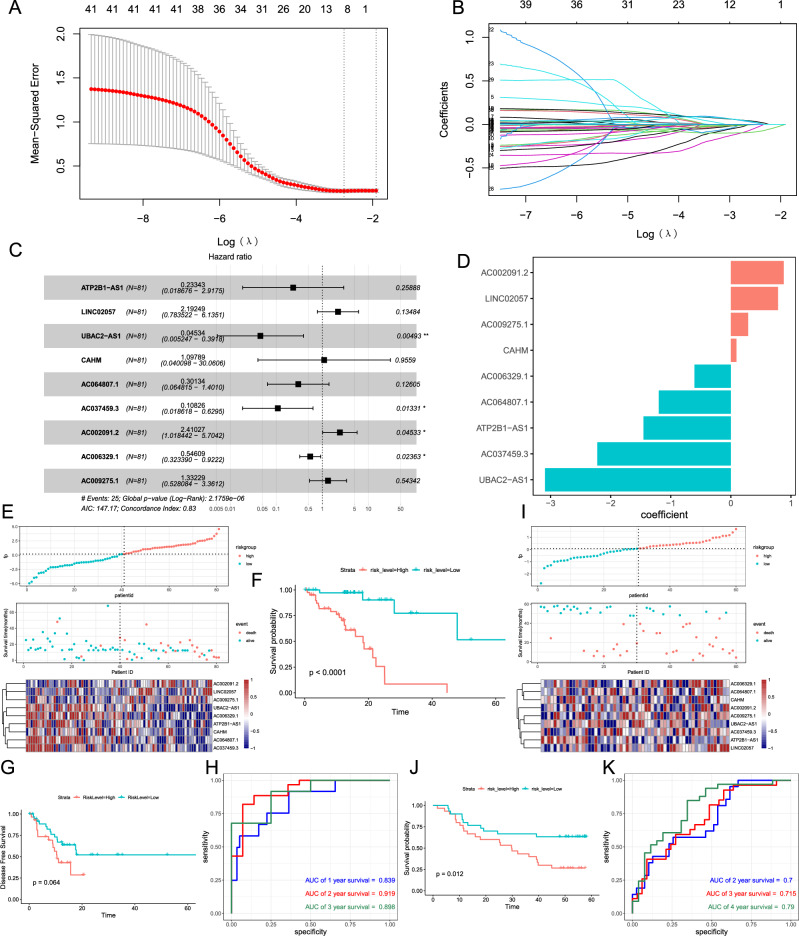
Identification and validation of the m^5^C-related lncRNAs prognostic signature (m^5^C-LPS) based on the cohort of TCGA-ESCC and GSE53622. **(A,B)** The minimum criterion of the LASSO regression algorithm was used to identify the most robust prognostic m^5^C-related lncRNAs. **(C)** Forest plot presenting the hazard ratio (HR) and 95% confidence interval (CI) of the 9 lncRNAs by the multivariate Cox regression. **(D)** The coefficients of the 9 lncRNAs contained in the m^5^C-LPS formula. **(E)** The distributions of the risk score, vital status, overall survival (OS), and expression levels of the 9 m^5^C-related lncRNAs in low- and high-risk groups in the cohort from TCGA-ESCC. **(F)** Kaplan–Meier (K–M) analysis demonstrated that patients with higher risk scores exhibited worse overall survival in the cohort from TCGA-ESCC. **(G)** K–M analysis demonstrated that patients with higher risk scores exhibited worse disease-free survival in the cohort from TCGA-ESCC. **(H)** The area under the curve (AUC) of the time-dependent ROC curves measures the predictive value of the risk score in the cohort from TCGA-ESCC. **(I)** The distributions of the risk score, vital status, overall survival (OS), and expression levels of the 9 m^5^C-related lncRNAs in low- and high-risk groups in the cohort from GSE53622. **(J)** K–M analysis demonstrated that patients with higher risk scores exhibited worse overall survival in the cohort from GSE53622. **(K)** AUC of the time-dependent ROC curves measuring the predictive value of the risk score in the cohort from GSE53622.

To eliminate the collinearity of variables and minimize estimation variance, LASSO regression analysis was applied to establish a prognostic signature using the 41 aforementioned lncRNAs. Subsequently, an m^5^C-LPS comprising 9 lncRNAs was identified based on the optimal λ value (Fig. 2A,B). Subsequently, the risk score was calculated based on the coefficients of the nine identified lncRNAs and their corresponding expression levels, yielding a concordance index (C-index) of 0.83, indicating strong discriminatory power (Fig. 2C,D). Besides, the model exhibited a sensitivity of 0.880, specificity of 0.643, positive likelihood ratio of 2.464, negative likelihood ratio of 0.187, positive predictive value of 0.524, and negative predictive value of 0.923. The m^5^C-LPS formula was calculated as follows: $\mathrm{Risk\, Score}=\left(-1.45488\right)\times ATP2B1-AS1+0.78504\times LINC02057+\left(-3.09357\right)\times UBAC2-AS1+0.09339\times CAHM+\left(-1.19951\right)\times AC064807.1+\left(-2.22323\right)\times AC037459.3+0.87974\times AC002091.2+\left(-0.60497\right)\times AC006329.1+0.2869\times AC009275.1.$

Subsequently, we categorized the 80 ESCC patients into low- and high-risk groups based on the median risk score. And the vital status and expression levels of the corresponding 9 lncRNAs in the cohort from TCGA-ESCC have presented in Fig. 2E. K–M analysis revealed that the patients in the high-risk group had relatively poorer OS and disease-free survival (DFS) compared with the low-risk group (OS: *p* < 0.0001, DFS: *p* = 0.064, Fig. 2F,G). Moreover, time-dependent ROC curves implied that m^5^C-LPS exhibited a promising ability to predict prognosis in the TCGA-ESCC cohort (1-year AUC = 0.839, 2-year AUC = 0.919, 3-year AUC = 0.898; Fig. 2H).

### Validation of m^5^C-LPS in the cohort from the GEO database

To validate the prognostic value of m^5^C-LPS, we calculated risk scores for another 60 ESCC patients from the GSE53622 cohort using the same formula. ESCC patients were divided into low- and high-risk groups according to the medium value. The distribution of the risk score, survival status, and lncRNAs expression showed that patients with higher risk scores had shorter OS and higher mortality status (Fig. 2I). Consistent with the findings in the TCGA-ESCC cohort, patients in the high-risk group presented significantly poorer prognoses (*p* = 0.012, Fig. 2J). And the AUC of the m^5^C-LPS was 0.7 at 2 years, 0.715 at 3 years, and 0.79 at 4 years (Fig. 2K).

### Co-expression status and differential expression of m^5^C-related lncRNAs

**Figure 3:**
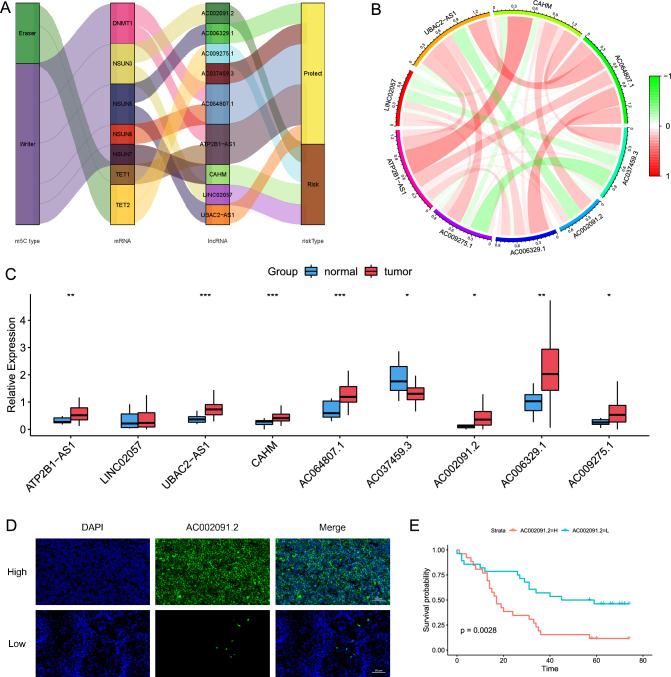
Co-expression status and expression level of m^5^C-related lncRNAs in TCGA-ESCC database and ESCC cell lines. **(A)** Sankey plot showing one-to-one matches between m^5^C genes, m^5^C-related lncRNAs, and their risk type. **(B)** Circle plot presenting the co-expression status of the 9 m^5^C-related lncRNAs with coefficients annotated. **(C)** The expression level of m^5^C-related lncRNAs in normal esophageal and ESCC tissues based on TCGA-ESCC database. **(D)** Representative Fluorescence in situ hybridization (FISH) images of *AC002091.2* in ESCC tissues. Scale bars represent 50 μm. **(E)** K–M plot for overall survival grouped by *AC002091.2* expression in 54 ESCC patients. *p < 0.05, **p < 0.01, ***p < 0.001.

We examined the co-expression status and differential expression of the 9 m^5^C-related lncRNAs. The Sankey plot showed one-to-one matches between the 7 m^5^C genes (5 writers: *DNMT1, NSUN3, NSUN5-7*; 2 erasers: *TET1-2*) and the 9 lncRNAs used in constructing the m^5^C-LPS. Additionally, the Sankey plot also depicted the risk type of each lncRNA (risk lncRNAs: *AC002091.2, AC009275.1, CAHM,* and *LINC02057.1*; protect lncRNAs: *AC0006329.1, AC037459.3, AC064807.1, ATP2B1-AS1*, and *UBAC2-AS1*, Fig. 3A). Moreover, the correlation circle plot revealed a general positive correlation among these m^5^C-related lncRNAs, except for *CHAM* and *AC037459.3* had negative relationship with *AC009275.1* and *LINC02057*, and *UBAC2-AS1* showed negative correlation with *AC002091.2* (Fig. 3B). Then, we compared the expression levels of these lncRNAs in normal esophageal and ESCC samples and observed that 7 lncRNAs were upregulated and 1 lncRNA was downregulated in ESCC samples (Fig. 3C).

Subsequently, we performed qRT-PCR using normal esophageal cell line HET-1A and ESCC cell lines TE-1 and KYSE150. The boxplot revealed that the upregulation of *LINC02057, UBAC2-AS1, CAHM, AC002091.2*, *AC006329.1,* and *AC009275.1* in ESCC cells, while *AC037459.3* was downregulated in ESCC cells (Fig. S3A). And we also detected the expression of these lncRNAs in ESCC and adjacent normal tissues and found that most of lncRNAs were upregulated in ESCC tissues (Fig. S3B). These results indicated that the expression patterns of m^5^C-related lncRNAs are consistent with the findings from the TCGA database. Since *AC002091.2* was upregulated in ESCC cell lines and tissues and was of great prognostic value for ESCC patients, we subsequently investigated the relationship between the expression of *AC002091.2* and patients’ survival. The FISH results showed that *AC002091.2* was located in the cytoplasm (Fig. 3D). And K–M plot revealed that patients with higher *AC002091.2* expression had relatively poor prognosis (Fig. 3E, *p* = 0.0028).

### Correlation of the risk score acquired from m^5^C-LPS and clinicopathological parameters

**Figure 4:**
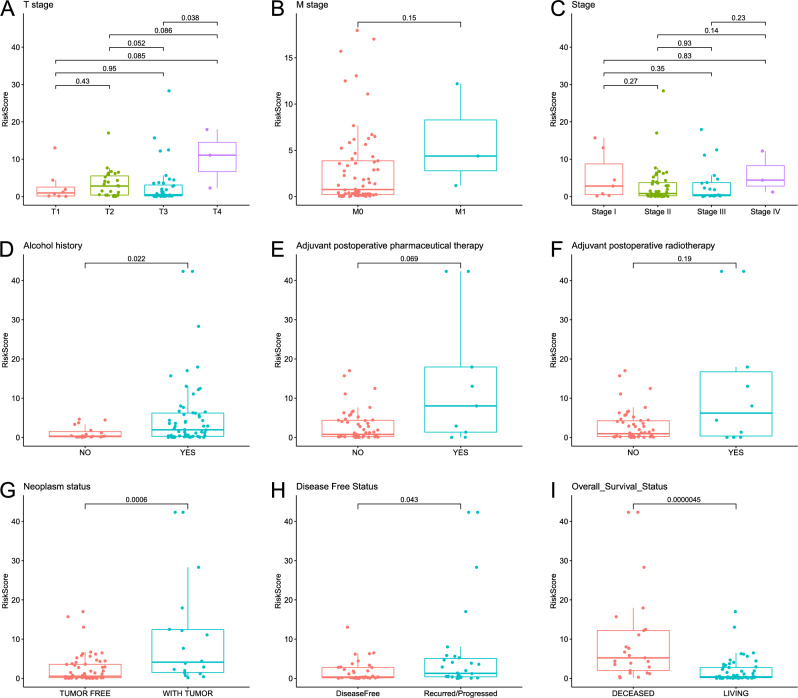
The discrepancy in risk scores between different subgroups: T stage **(A)**, M stage **(B)**, stage **(C)**, alcohol history **(D),** adjuvant postoperative pharmaceutical therapy **(E)**, adjuvant postoperative radiotherapy **(F)**, neoplasm status **(G)**, disease free status **(H)**, and overall survival status (**I**).

To evaluate the clinical significance of m^5^C-LPS, we assessed its association with various clinicopathological parameters of ESCC. Subgroup analysis stratified by T stage revealed a significantly higher risk score in T4 ESCC patients compared to T3 ESCC patients (*p* = 0.038, Fig. 4A). Stratification by M stage indicated an increased risk score in M1 patients, although the difference did not reach statistical significance (*p* = 0.15, Fig. 4B). No significant differences were observed between age, gender, race, tumor location, histologic grade, N stage, stage, reflux history and risk score (*p* > 0.05, Fig. S4A–G, Fig. 4C). Besides, ESCC patients with alcohol history exhibited a significantly elevated risk score than those without alcohol history (*p* = 0.022, Fig. 4D), while ESCC patients with or without smoking history had similar risk score (*p* = 0.71, Fig. S4H). In subgroup analysis stratified by adjuvant postoperative therapy, there was a trend towards a higher risk score in the pharmaceutical therapy and radiotherapy subgroup, although statistical significance was not achieved (*p* = 0.069 and 0.19, Fig. 4E,F). And ESCC patients with or without complete response after radiotherapy exhibited comparable risk scores (*p* = 0.47, Fig. S4I). Furthermore, ESCC patients with tumor presence, recurred/progressed, and deceased status had significantly increased risk scores (*p* < 0.05, Fig. 4G–I), consistent with previous results highlighting the value of m^5^C-LPS as a valuable prognostic marker.

### Evaluation of the prognostic value of m^5^C-LPS and construction of a nomogram

**Figure 5:**
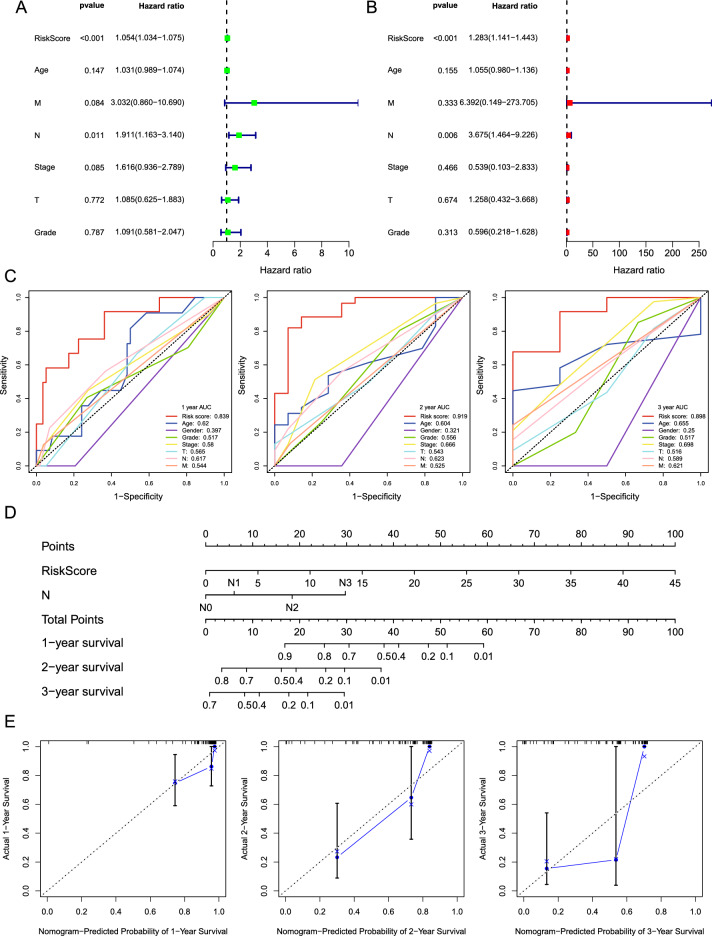
Verification of the independent prognostic value of m^5^C-LPS and construction of nomogram. Univariate **(A)** and multivariate **(B)** Cox regression analyses of the prognostic value of risk scores and other clinical parameters. **(C)** The ROC curves show the predictive value of the risk score and other clinical characteristics. **(D)** Nomogram composed of N stage and risk score was constructed to predict 1-, 2-, and 3-year survival rates. **(E)** Calibration plots were used to evaluate the nomogram for predicting 1-, 2-, and 3-year survival rates.

Univariate and multivariate Cox regression analyses were conducted to determine the independent prognostic value of m^5^C-LPS and other clinicopathological parameters for ESCC patients. The forest plots showed that the N stage and risk score were independent factors for the poor prognosis (*p* < 0.05, Fig. 5A,B). Subsequently, we used time-dependent ROC curves to evaluate the prognostic potential of the risk score, age, gender, grade, stage, and TNM stage. The AUC values of the risk score were higher than those of other clinicopathological factors for 1-, 2-, and 3-year survival (Fig. 5C). These findings highlight the significant value of the risk score in predicting patient prognosis. Meanwhile, a nomogram was constructed based on the risk score and N stage of each ESCC patient, which could be a quantitative tool to predict 1-, 2-, and 3-year survival probability (Fig. 5D). Moreover, the calibration curves showed partial agreement between the predicted and observed survival probabilities (Fig. 5E).

### Exploration of immune microenvironment affected by m^5^C-LPS

**Figure 6:**
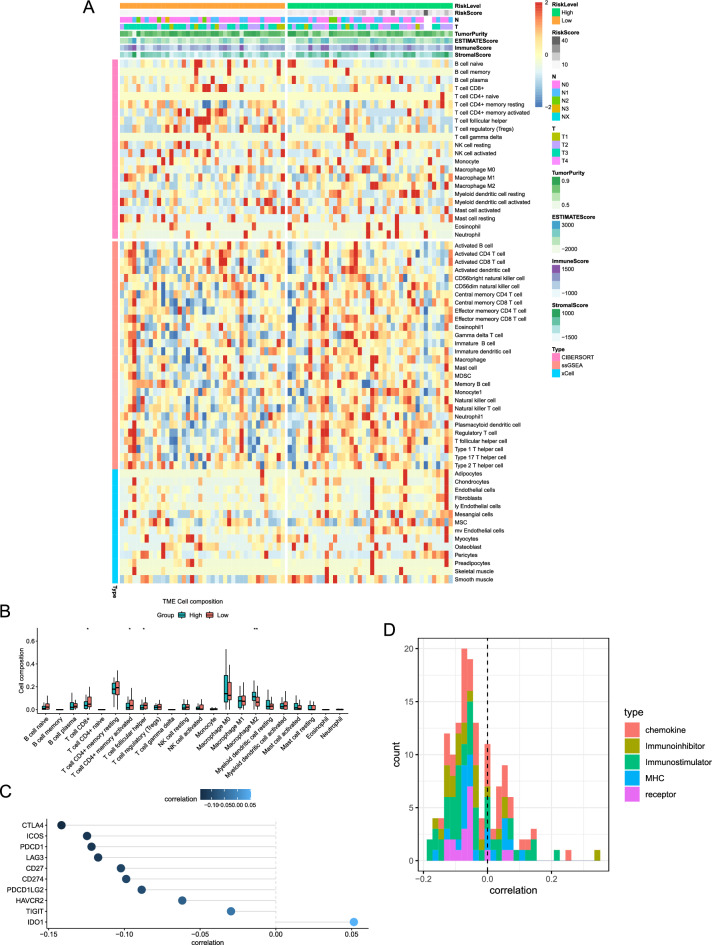
Investigation of immune status in different risk groups. **(A)** Heatmap revealing the immune and stromal cells infiltration in ESCC immune microenvironment. **(B)** Box plots showing the infiltration of the immune cells based on the Cibersort algorithm in different risk groups; *p < 0.05 and **p < 0.01. **(C)** Estimation of the coefficients for risk score with immune checkpoint genes. **(D)** Histogram showing the relationships between risk score and chemokines, receptors, MHC, immunoinhibitors, and immunostimulators.

We further investigated the relationship between the immune microenvironment and the risk score obtained from m^5^C-LPS. The relative abundance of immune and stromal cells of each sample was estimated using Estimate, Cibersort, ssGSEA, and xCELL algorithms. The heatmap revealed the different distribution patterns of various cell types between the low- and high-risk groups (Fig. 6A). Comparison of the Cibersort results revealed significant enrichments of CD8^+^ T cells, memory activated CD4^+^ T cells, and T follicular helper cells in the low-risk group, while M2 macrophages were found to be enriched in the high-risk group (Fig. 6B). The ssGSEA results showed that central memory CD8^+^ T cell, gamma delta T cell, macrophage, NK cell, plasmacytoid dendritic cell, Tregs, and T follicular helper cell were significantly enriched in the high-risk group (*p* < 0.05, Fig. S5A). However, there were no significant differences in stromal cells between the low- and high-risk groups (Fig. S5B). The correlation heatmap identified three main clustering modules: function immune cells, resting immune cells, and stromal cells (Fig. S6A). Furthermore, the correlation coefficient indicated a negative association between the risk scores and multiple well-known immune checkpoint molecules, except for IDO1 (Fig. 6C). The histogram and heatmap revealed inverse relationships between the risk score and most immunomodulators, including chemokines, receptors, MHC, immunoinhibitors, and immunostimulators (Fig. 6D, Fig. S6B). These findings indicated that the activation of immune components in the tumor microenvironment may contribute to better outcomes for patients in the low-risk group.

### Comprehensive analysis of enriched pathways between different risk groups

To elucidate the biological functions of the differentially expressed genes associated with m^5^C-LPS, we performed GO, KEGG, and Reactome enrichment analyses. The prominent GO terms in molecular function (MF), cellular component (CC), and biological process (BP) were catalytic activity acting on RNA, nuclear speck, and ncRNA metabolic process, respectively (Fig. S7A). Furthermore, the top five enriched KEGG terms included spliceosome, cell cycle, ribosome biogenesis in eukaryotes, RNA degradation, and Homologous recombination (Fig. S7B). The three main key modules identified in the Reactome analysis were rRNA processing, mRNA splicing and processing, and DNA damage repair response (Fig. S7C).

### The genomic alteration difference between two m^5^C-LPS groups

By analyzing the MuTect2 mutation annotation files, we identified the top 20 most frequently mutated genes in the low- and high-risk groups, as illustrated in Fig. S8A,B, respectively. The waterfall plots revealed that *TP53*, *TTN*, and *KMT2D* were most frequently mutated in both groups. However, the ranking of mutated genes showed slight changes between the two groups. For example, the mutation frequency of *MUC16* was ranked third in the high-risk group (20%), but it dropped out of the top 20 mutated genes in the low-risk group. Furthermore, the mutation rates of seven oncogenic pathways (*NOTCH, WNT, PI3K, MYC, TP53*, *TGF-beta*, Cell-Cycle) were higher in the high-risk group compared to the low-risk group (Fig. S8C,D). These findings suggested that ESCC patients in the low- and high-risk groups may have different mutation driver genes and pathways.

### m^5^C-LPS predict therapeutic response in ESCC patients

**Figure 7:**
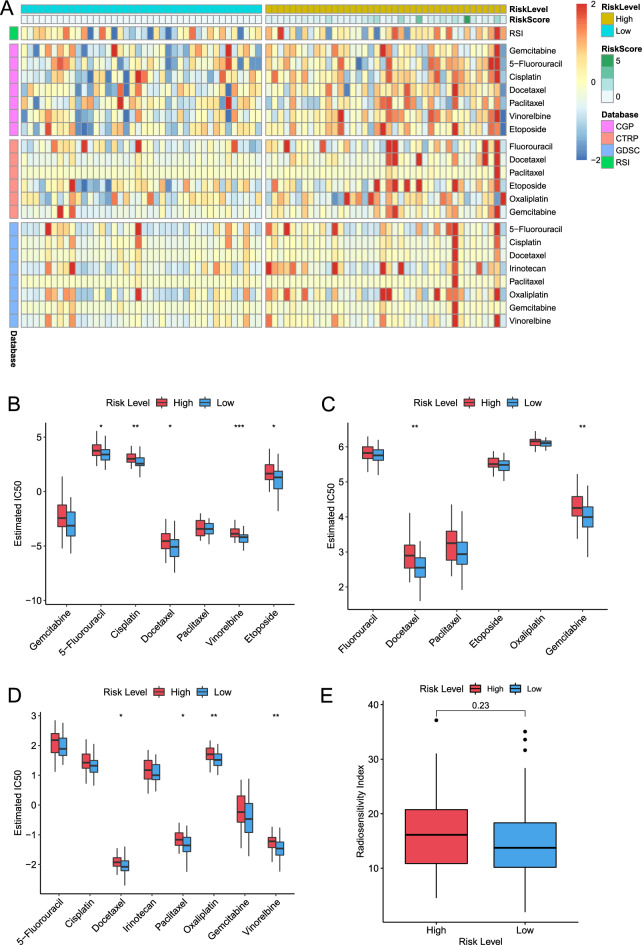
Identification of therapeutic response features of different risk groups. **(A)** Heatmap revealing IC50 for chemotherapeutic agents and radiation-sensitivity index (RSI) for radiotherapy. Box plots showing the sensitivity of selected chemotherapeutic agents for patients in low- and high-risk groups based on CGP **(B)**, CTRP **(C)**, and GDSC **(D)** databases. **(E)** Boxplot showing the radiotherapy sensitivity for patients in low- and high-risk groups.

Given that chemotherapy and radiotherapy are crucial in ESCC treatments, and DNA damage repair response plays a pivotal role in regulating chemoradiotherapy response, we attempted to evaluate the therapeutic response of the low- and high-risk groups. We estimated the IC50 levels of several commonly used chemotherapeutic drugs in each patient using CGP, CTRP, and GDSC-derived drug response data. The heatmap showed that the estimated IC50 levels of these drugs were reduced in the low-risk group, indicating that patients in low-risk group were more sensitive to chemotherapy (Fig. 7A). Boxplots further demonstrated that patients in the low-risk group exhibited greater sensitivity to five CGP-derived compounds (5-fluorouracil, cisplatin, docetaxel, vinorelbine, and etoposide), two CTRP-derived compounds (docetaxel and gemcitabine), and four GDSC-derived compounds (docetaxel, paclitaxel, oxaliplatin, and vinorelbine). And significant differences in the IC50 level of docetaxel were observed among three database-derived results (Fig. 7B–D). Besides, the radiation-sensitivity index (RSI) increased in the high-risk group, suggesting that patients in the high-risk group might require a higher radiotherapy dose, although there was no statistical significance (*p* = 0.23, Fig. 7E).

## Discussion

ESCC accounts for about 90% of the incidence of EC annually with a dismal 5-year survival rate of 5–25% worldwide^1,3,43^. To date, molecular-related target therapy had emerged as new therapeutic strategies for prolonging patients’ prognosis. In recent years, RNA post-transcriptional methylation modification, including m^6^A, m^5^C, and m^1^A, has arrested substantial attention among researchers worldwide^44,45^. Over the past decade, numerous m^5^C regulators have been found to play pivotal roles in regulating gene expression and disease progression, including cancer^46,47^. For instance, *NSUN2*, which plays crucial roles in tissue homeostasis, spindle stability, and early embryogenesis as a nucleolar protein, is overexpressed and possesses prognostic survival value in various tumors^48,49^. While the function of m^5^C modification in other cancers has been extensively studied^12,50,51^, its effect on ESCC has not been fully explored. In the present study, we observed the upregulation of 11 m^5^C regulators in ESCC tissues compared to normal adjacent tissues (Fig. 1A). Thus, we aimed to investigate the role of m^5^C in ESCC further.

Existing evidences have testified that m^5^C methylated lncRNA can regulate the occurrence and development of cancer^20^. The “writer” *NSUN2* modifies the lncRNA *H19* and recruits the oncoprotein *G3BP1* in hepatocellular carcinoma, suggesting that m^5^C modifications are involved in malignant tumor progression^52^. Furthermore, as dysregulation of lncRNAs plays a crucial role in tumor development, and they can be detected in easily accessible bodily fluids like urine, saliva, and serum, they have great potential as prognostic biomarkers and therapeutic targets for tumors^53^. We believe that investigating the interplay between m^5^C regulators and lncRNAs will become a promising area for identifying prognostic markers and therapeutic targets for cancers. Nonetheless, the role of lncRNAs involved in m^5^C regulation in ESCC remains unclear. To our knowledge, this is the first comprehensive analysis of the function of m^5^C-related lncRNAs in ESCC.

In this study, we evaluated the prognostic value of m^5^C-related lncRNAs in ESCC patients. A prognostic model based on 9 m^5^C-related lncRNAs was constructed using univariate and LASSO Cox regression analyses, and a formula for the calculation of risk score was established. The prognostic value of m^5^C-LPS was then tested in both training (TCGA-ESCC) and validation (GSE53622) datasets (Fig. 2). These results suggest that m^5^C-LPS could serve as a powerful tool for predicting the prognosis of ESCC patients.

Limited information is currently available on the lncRNAs identified in our study. However, the functions that have been reported for *CAHM*, *ATP2B1-AS1*, and *UBAC2-AS1* provide important insights into the potential roles of these 9 novel m^5^C-related lncRNAs. The well-established functions of *CAHM*, which is also known as colorectal adenocarcinoma hypermethylated, as a prognostic biomarker in colorectal and thyroid carcinoma^54,55^, and its regulation by *DNMT1* in glioma cells, suggest its involvement in glioma grade, subtype, malignant behavior, and prognosis^56^. Similarly, the involvement of *ATP2B1-AS1* in the NF-kappa-B signaling pathway, which plays a crucial role in tumorigenesis, particularly in gastrointestinal cancers^57,58^, suggests its potential as a target for therapeutic intervention. Furthermore, the close association between *UBAC2-AS1* and autophagy genes highlights its potential involvement in cancer-related processes and its possible therapeutic implications^59^. Our study has revealed the overexpression of these lncRNAs in ESCC. However, further research is needed to elucidate the precise functions and mechanisms of these lncRNAs. Nonetheless, our study provides a foundation for exploring these lncRNAs as potential therapeutic targets in cancer treatment.

Due to the association between m^5^C-LPS and immune status being weak, we further investigated the signaling pathways and biological functions related to m^5^C-LPS. Our analysis revealed a significant enrichment of functions associated with mRNA and ncRNA processing, as well as DNA damage repair response. These findings align with the established functions of m^5^C and lncRNAs previously reported in the literature^14,60–62^. For instance, the mRNA and translation levels enhanced when *NSUN6*-targeted mRNAs were methylated^63^. *TRDMT1–FMRP–TET1*-mediated m^5^C regulation can promote transcription-coupled homologous recombination^64^. And *TRDMT1* can mediate m^5^C mRNA methylation at DNA damage sites and regulate homologous recombination^60^. Besides, DNA damage repair response can regulate the response effectiveness of chemoradiotherapy^65,66^, which is the mainstay for ESCC treatment^43,67^, we evaluated the therapeutic response of ESCC patients in the TCGA-ESCC cohort. Our analysis demonstrated that patients in the low-risk group exhibited a higher sensitivity to chemoradiotherapy. Additionally, studies in leukemia have shown that *NSUN3* and *DNMT2* can regulate the chromatin structures by directly binding *hnRNPK* and further modulating 5-Azacitidine response^68^. These observations provide valuable insights into the potential role of m^5^C-LPS as a predictive marker and highlight the need for further exploration of m^5^C function in cancer treatment.

This study has several limitations that should be acknowledged. Firstly, the small sample size, retrospective nature, and non-uniform patient source and race of the TCGA-ESCC and GSE53622 cohorts may have influenced the results. And the absence of an independent clinical cohort limits the validation of the prognostic signature. Thus, more high-quality cohort data are needed in the future to validate the prognostic value and chemoradiotherapy response of m^5^C-LPS. Secondly, although we detected the expression of the 9 identified lncRNAs in m^5^C-LPS in ESCC and normal esophageal cell lines, further in vitro and in vivo experiments are required to support our in silico results.

In this study, we constructed and validated a prognostic signature based on 9 m^5^C-related lncRNAs for ESCC patients. And we found that stratification of ESCC patients based on m^5^C-LPS is associated with different clinical features, immune status, genomic variants, oncogenic pathways, enrichment pathways, and therapeutic responses. In summary, our study provides a valuable tool for understanding the potential role of m^5^C-related lncRNAs and guiding personalized management of ESCC.

### Supplementary Information

Supplementary Figure S1.

Supplementary Figure S2.

Supplementary Figure S3.

Supplementary Figure S4.

Supplementary Figure S5.

Supplementary Figure S6.

Supplementary Figure S7.

Supplementary Figure S8.

Supplementary Information.

## Data Availability

The data presented in this study can be found in TCGA (https://portal.gdc.cancer.gov/repository?facetTab=cases) and GSE53622 (https://www.ncbi.nlm.nih.gov/geo/query/acc.cgi?acc=gse53622) databases.

## Abbreviations

AUC: Area under the curve
ESCC: Esophageal squamous cell carcinoma
CGP: Cancer Genome Project
CTRP: Cancer therapeutics response portal
GARD: Genomic-adjusted radiation dose
GDSC: Genomics of drug sensitivity in cancer
GO: Gene ontology
HPA: Human Protein Atlas
IC50: Half-maximal inhibitory concentration
KEGG: Kyoto Encyclopedia of Genes and Genomes
K–M: Kaplan–Meier
LASSO: Least absolute shrinkage and selection operator
lncRNAs: Long non-coding RNA
m^5^C: 5-Methylcytosine
m^5^C-LPS: m^5^C-Related lncRNAs prognostic signature
OS: Overall survival
PPI: Protein–protein interaction
qRT-PCR: Quantitative real-time polymerase chain reaction
ROC: Receiver operating characteristic
ssGSEA: Single-sample gene set enrichment analysis
TCGA: The Cancer Genome Atlas

## References

[1] Sung H, Ferlay J, Siegel RL, Laversanne M, Soerjomataram I, Jemal A, Bray F (2021). Global cancer statistics 2020: GLOBOCAN estimates of incidence and mortality worldwide for 36 cancers in 185 countries. CA Cancer J. Clin..

[2] Arnold M, Ferlay J, van Berge Henegouwen MI, Soerjomataram I (2020). Global burden of oesophageal and gastric cancer by histology and subsite in 2018. Gut.

[3] Abnet CC, Arnold M, Wei WQ (2018). Epidemiology of esophageal squamous cell carcinoma. Gastroenterology.

[4] Thrift AP (2021). Global burden and epidemiology of Barrett oesophagus and oesophageal cancer. Nat. Rev. Gastroenterol. Hepatol..

[5] Waters JK, Reznik SI (2022). Update on management of squamous cell esophageal cancer. Curr. Oncol. Rep..

[6] Zugazagoitia J, Guedes C, Ponce S, Ferrer I, Molina-Pinelo S, Paz-Ares L (2016). Current challenges in cancer treatment. Clin. Ther..

[7] Zhang Q, Liu F, Chen W, Miao H, Liang H, Liao Z, Zhang Z, Zhang B (2021). The role of RNA m(5)C modification in cancer metastasis. Int. J. Biol. Sci..

[8] Haruehanroengra P, Zheng YY, Zhou Y, Huang Y, Sheng J (2020). RNA modifications and cancer. RNA Biol..

[9] Su J, Wu G, Ye Y, Zhang J, Zeng L, Huang X, Zheng Y, Bai R, Zhuang L, Li M (2021). NSUN2-mediated RNA 5-methylcytosine promotes esophageal squamous cell carcinoma progression via LIN28B-dependent GRB2 mRNA stabilization. Oncogene.

[10] Murata A, Baba Y, Ishimoto T, Miyake K, Kosumi K, Harada K, Kurashige J, Iwagami S, Sakamoto Y, Miyamoto Y (2015). TET family proteins and 5-hydroxymethylcytosine in esophageal squamous cell carcinoma. Oncotarget.

[11] Zhou M, Liu W, Zhang J, Sun N (2021). RNA m(6)A modification in immunocytes and DNA repair: The biological functions and prospects in clinical application. Front. Cell. Dev. Biol..

[12] Guo G, Pan K, Fang S, Ye L, Tong X, Wang Z, Xue X, Zhang H (2021). Advances in mRNA 5-methylcytosine modifications: Detection, effectors, biological functions, and clinical relevance. Mol. Ther. Nucleic Acids.

[13] García-Vílchez R, Sevilla A, Blanco S (2019). Post-transcriptional regulation by cytosine-5 methylation of RNA. Biochim. Biophys. Acta Gene Regul. Mech..

[14] Bohnsack KE, Höbartner C, Bohnsack MT (2019). Eukaryotic 5-methylcytosine (m5C) RNA methyltransferases: Mechanisms, cellular functions, and links to disease. Genes.

[15] Ransohoff JD, Wei Y, Khavari PA (2018). The functions and unique features of long intergenic non-coding RNA. Nat. Rev. Mol. Cell Biol..

[16] Fang Y, Fullwood MJ (2016). Roles, functions, and mechanisms of long non-coding RNAs in cancer. Genom. Proteom. Bioinform..

[17] Zhang X, Xie K, Zhou H, Wu Y, Li C, Liu Y, Liu Z, Xu Q, Liu S, Xiao D (2020). Role of non-coding RNAs and RNA modifiers in cancer therapy resistance. Mol. Cancer.

[18] Liang Y, Chen X, Wu Y, Li J, Zhang S, Wang K, Guan X, Yang K, Bai Y (2018). LncRNA CASC9 promotes esophageal squamous cell carcinoma metastasis through upregulating LAMC2 expression by interacting with the CREB-binding protein. Cell Death Differ..

[19] Torsin LI, Petrescu GED, Sabo AA, Chen B, Brehar FM, Dragomir MP, Calin GA (2021). Editing and chemical modifications on non-coding RNAs in cancer: A new tale with clinical significance. Int. J. Mol. Sci..

[20] He Y, Shi Q, Zhang Y, Yuan X, Yu Z (2020). Transcriptome-wide 5-methylcytosine functional profiling of long non-coding RNA in hepatocellular carcinoma. Cancer Manag. Res..

[21] Squires JE, Patel HR, Nousch M, Sibbritt T, Humphreys DT, Parker BJ, Suter CM, Preiss T (2012). Widespread occurrence of 5-methylcytosine in human coding and non-coding RNA. Nucleic Acids Res..

[22] Li Y, Li J, Luo M, Zhou C, Shi X, Yang W, Lu Z, Chen Z, Sun N, He J (2018). Novel long noncoding RNA NMR promotes tumor progression via NSUN2 and BPTF in esophageal squamous cell carcinoma. Cancer Lett..

[23] Szklarczyk D, Gable AL, Nastou KC, Lyon D, Kirsch R, Pyysalo S, Doncheva NT, Legeay M, Fang T, Bork P (2021). The STRING database in 2021: Customizable protein–protein networks, and functional characterization of user-uploaded gene/measurement sets. Nucleic Acids Res..

[24] Tibshirani R (1997). The lasso method for variable selection in the Cox model. Stat. Med..

[25] Ternès N, Rotolo F, Michiels S (2016). Empirical extensions of the lasso penalty to reduce the false discovery rate in high-dimensional Cox regression models. Stat. Med..

[26] Pak K, Oh SO, Goh TS, Heo HJ, Han ME, Jeong DC, Lee CS, Sun H, Kang J, Choi S (2020). A user-friendly, web-based integrative tool (ESurv) for survival analysis: Development and validation study. J. Med. Internet Res..

[27] Consortium GO (2021). The Gene Ontology resource: Enriching a GOld mine. Nucleic Acids Res..

[28] Kanehisa M, Furumichi M, Tanabe M, Sato Y, Morishima K (2017). KEGG: New perspectives on genomes, pathways, diseases and drugs. Nucleic Acids Res..

[29] Jassal B, Matthews L, Viteri G, Gong C, Lorente P, Fabregat A, Sidiropoulos K, Cook J, Gillespie M, Haw R (2020). The reactome pathway knowledgebase. Nucleic Acids Res..

[30] Yu G, Wang LG, Han Y, He QY (2012). clusterProfiler: An R package for comparing biological themes among gene clusters. OMICS.

[31] Yoshihara K, Shahmoradgoli M, Martínez E, Vegesna R, Kim H, Torres-Garcia W, Treviño V, Shen H, Laird PW, Levine DA (2013). Inferring tumour purity and stromal and immune cell admixture from expression data. Nat. Commun..

[32] Hänzelmann S, Castelo R, Guinney J (2013). GSVA: Gene set variation analysis for microarray and RNA-seq data. BMC Bioinform..

[33] Newman AM, Liu CL, Green MR, Gentles AJ, Feng W, Xu Y, Hoang CD, Diehn M, Alizadeh AA (2015). Robust enumeration of cell subsets from tissue expression profiles. Nat. Methods.

[34] Aran D, Hu Z, Butte AJ (2017). xCell: Digitally portraying the tissue cellular heterogeneity landscape. Genome Biol..

[35] Ru B, Wong CN, Tong Y, Zhong JY, Zhong SSW, Wu WC, Chu KC, Wong CY, Lau CY, Chen I (2019). TISIDB: An integrated repository portal for tumor-immune system interactions. Bioinformatics.

[36] Mayakonda A, Lin DC, Assenov Y, Plass C, Koeffler HP (2018). Maftools: Efficient and comprehensive analysis of somatic variants in cancer. Genome Res..

[37] Sanchez-Vega F, Mina M, Armenia J, Chatila WK, Luna A, La KC, Dimitriadoy S, Liu DL, Kantheti HS, Saghafinia S (2018). Oncogenic signaling pathways in the cancer genome atlas. Cell.

[38] Geeleher P, Cox N, Huang RS (2014). pRRophetic: An R package for prediction of clinical chemotherapeutic response from tumor gene expression levels. PLoS ONE.

[39] Garnett MJ, Edelman EJ, Heidorn SJ, Greenman CD, Dastur A, Lau KW, Greninger P, Thompson IR, Luo X, Soares J (2012). Systematic identification of genomic markers of drug sensitivity in cancer cells. Nature.

[40] Rees MG, Seashore-Ludlow B, Cheah JH, Adams DJ, Price EV, Gill S, Javaid S, Coletti ME, Jones VL, Bodycombe NE (2016). Correlating chemical sensitivity and basal gene expression reveals mechanism of action. Nat. Chem. Biol..

[41] Yang W, Soares J, Greninger P, Edelman EJ, Lightfoot H, Forbes S, Bindal N, Beare D, Smith JA, Thompson IR (2013). Genomics of drug sensitivity in cancer (GDSC): A resource for therapeutic biomarker discovery in cancer cells. Nucleic Acids Res..

[42] Scott JG, Berglund A, Schell MJ, Mihaylov I, Fulp WJ, Yue B, Welsh E, Caudell JJ, Ahmed K, Strom TS (2017). A genome-based model for adjusting radiotherapy dose (GARD): A retrospective, cohort-based study. Lancet Oncol..

[43] Leng XF, Daiko H, Han YT, Mao YS (2020). Optimal preoperative neoadjuvant therapy for resectable locally advanced esophageal squamous cell carcinoma. Ann. N. Y. Acad. Sci..

[44] Zhao BS, Roundtree IA, He C (2017). Post-transcriptional gene regulation by mRNA modifications. Nat. Rev. Mol. Cell Biol..

[45] Gilbert WV, Bell TA, Schaening C (2016). Messenger RNA modifications: Form, distribution, and function. Science.

[46] Chen YS, Yang WL, Zhao YL, Yang YG (2021). Dynamic transcriptomic m(5) C and its regulatory role in RNA processing. Wiley Interdiscip. Rev. RNA.

[47] Wood S, Willbanks A, Cheng JX (2021). The role of RNA modifications and RNA-modifying proteins in cancer therapy and drug resistance. Curr. Cancer Drug Targets.

[48] Blanco S, Frye M (2014). Role of RNA methyltransferases in tissue renewal and pathology. Curr. Opin. Cell Biol..

[49] Chellamuthu A, Gray SG (2020). The RNA methyltransferase NSUN2 and its potential roles in cancer. Cells.

[50] Yin H, Huang Z, Niu S, Ming L, Jiang H, Gu L, Huang W, Xie J, He Y, Zhang C (2022). 5-Methylcytosine (m(5)C) modification in peripheral blood immune cells is a novel non-invasive biomarker for colorectal cancer diagnosis. Front. Immunol..

[51] Pan J, Huang Z, Xu Y (2021). m5C-related lncRNAs predict overall survival of patients and regulate the tumor immune microenvironment in lung adenocarcinoma. Front. Cell. Dev. Biol..

[52] Sun Z, Xue S, Zhang M, Xu H, Hu X, Chen S, Liu Y, Guo M, Cui H (2020). Aberrant NSUN2-mediated m(5)C modification of H19 lncRNA is associated with poor differentiation of hepatocellular carcinoma. Oncogene.

[53] Sarfi M, Abbastabar M, Khalili E (2019). Long noncoding RNAs biomarker-based cancer assessment. J. Cell Physiol..

[54] Pedersen SK, Mitchell SM, Graham LD, McEvoy A, Thomas ML, Baker RT, Ross JP, Xu ZZ, Ho T, LaPointe LC (2014). CAHM, a long non-coding RNA gene hypermethylated in colorectal neoplasia. Epigenetics.

[55] Xiao Y, Tu Y, Li Y (2021). Expression level of long non-coding RNA colon adenocarcinoma hypermethylated serves as a novel prognostic biomarker in patients with thyroid carcinoma. Biosci. Rep..

[56] Xu Y, Li Z, Huai T, Huo X, Wang H, Bian E, Zhao B (2021). DNMT1 mediated CAHM repression promotes glioma invasion via SPAK/JNK pathway. Cell. Mol. Neurobiol..

[57] Hoesel B, Schmid JA (2013). The complexity of NF-κB signaling in inflammation and cancer. Mol. Cancer.

[58] Peng C, Ouyang Y, Lu N, Li N (2020). The NF-κB signaling pathway, the microbiota, and gastrointestinal tumorigenesis: Recent advances. Front. Immunol..

[59] Jiang Q, Xue D, Shi F, Qiu J (2021). Prognostic significance of an autophagy-related long non-coding RNA signature in patients with oral and oropharyngeal squamous cell carcinoma. Oncol. Lett..

[60] Chen H, Yang H, Zhu X, Yadav T, Ouyang J, Truesdell SS, Tan J, Wang Y, Duan M, Wei L (2020). m(5)C modification of mRNA serves a DNA damage code to promote homologous recombination. Nat. Commun..

[61] Su M, Wang H, Wang W, Wang Y, Ouyang L, Pan C, Xia L, Cao D, Liao Q (2018). LncRNAs in DNA damage response and repair in cancer cells. Acta Biochim. Biophys. Sin. (Shanghai).

[62] Li M, Tao Z, Zhao Y, Li L, Zheng J, Li Z, Chen X (2022). 5-methylcytosine RNA methyltransferases and their potential roles in cancer. J. Transl. Med..

[63] Selmi T, Hussain S, Dietmann S, Heiß M, Borland K, Flad S, Carter JM, Dennison R, Huang YL, Kellner S (2021). Sequence- and structure-specific cytosine-5 mRNA methylation by NSUN6. Nucleic Acids Res..

[64] Yang H, Wang Y, Xiang Y, Yadav T, Ouyang J, Phoon L, Zhu X, Shi Y, Zou L, Lan L (2022). FMRP promotes transcription-coupled homologous recombination via facilitating TET1-mediated m5C RNA modification demethylation. Proc. Natl. Acad. Sci. U.S.A..

[65] O’Connor MJ (2015). Targeting the DNA damage response in cancer. Mol. Cell.

[66] Huang RX, Zhou PK (2020). DNA damage response signaling pathways and targets for radiotherapy sensitization in cancer. Signal Transduct Target Ther..

[67] Sasaki Y, Kato K (2016). Chemoradiotherapy for esophageal squamous cell cancer. Jpn. J. Clin. Oncol..

[68] Cheng JX, Chen L, Li Y, Cloe A, Yue M, Wei J, Watanabe KA, Shammo JM, Anastasi J, Shen QJ (2018). RNA cytosine methylation and methyltransferases mediate chromatin organization and 5-azacytidine response and resistance in leukaemia. Nat. Commun..

