# Supplementary figures and images for: Identification of m5C-related lncRNAs signature to predict prognosis and therapeutic responses in esophageal squamous cell carcinoma patients
Source: Sci Rep. 2023 Sep 4;13:14499. doi: 10.1038/s41598-023-41495-6 (PMC10477299; doi:10.1038/s41598-023-41495-6)

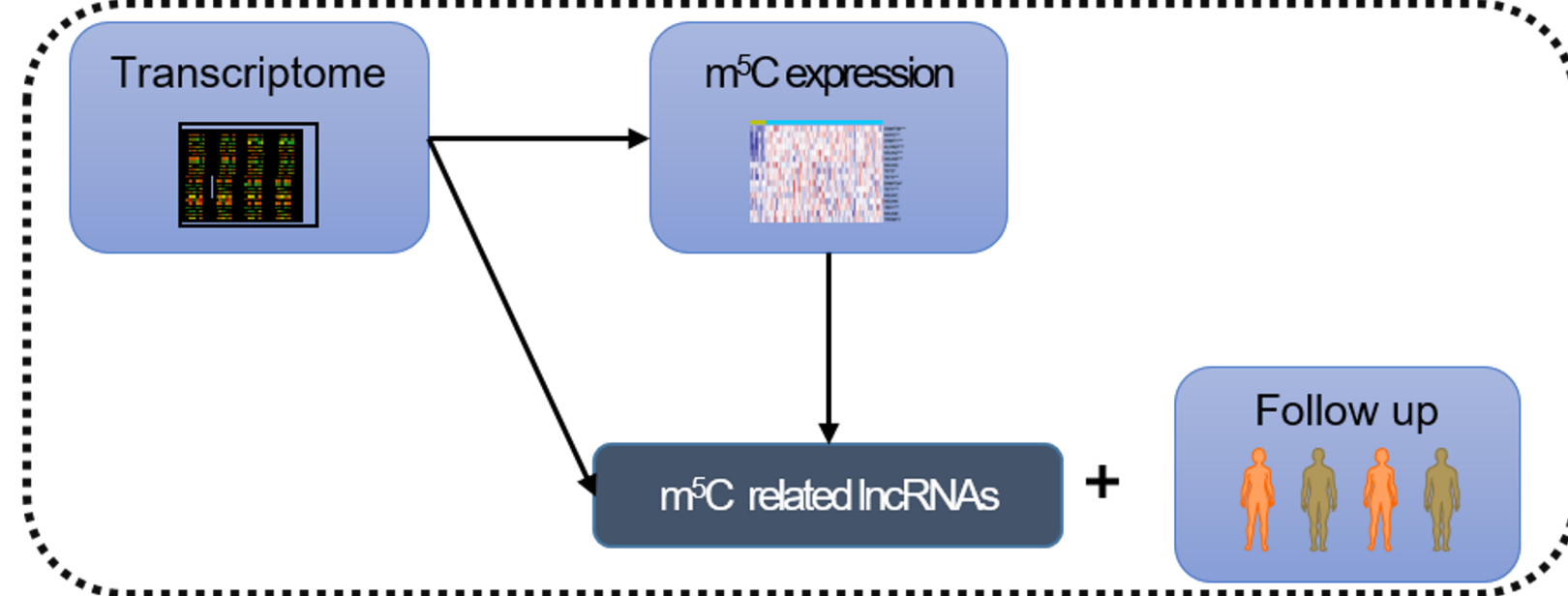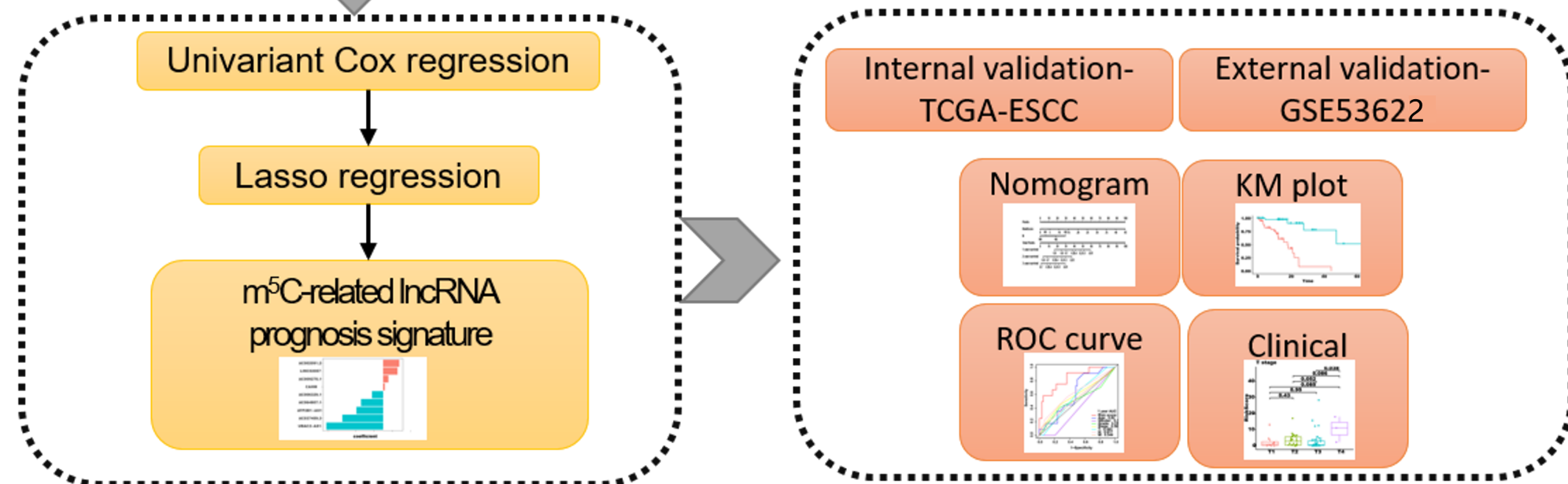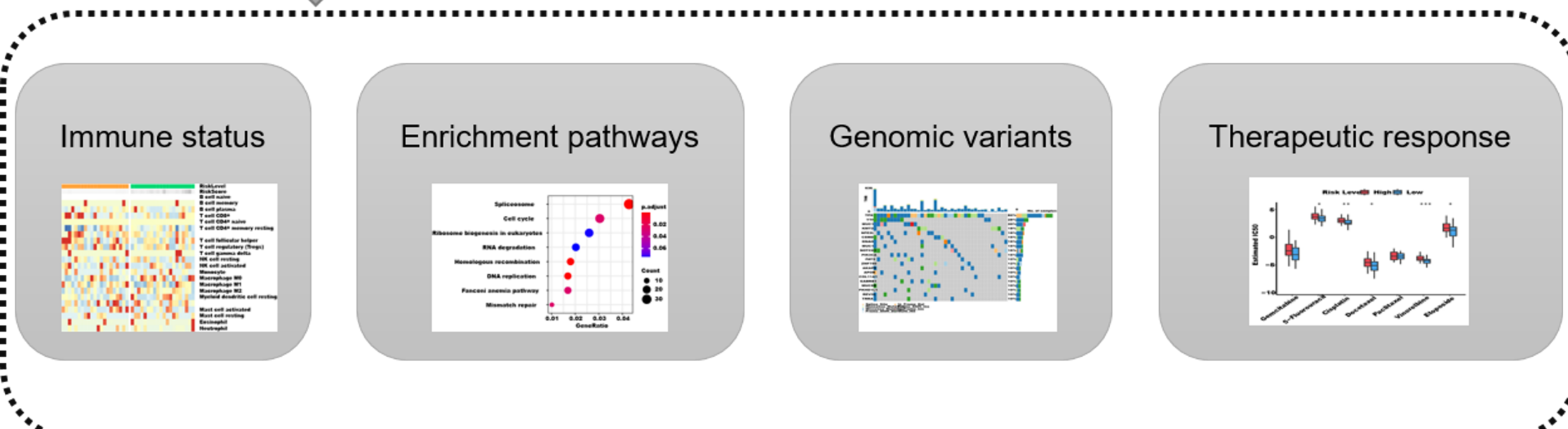

Supplement: Supplementary file 1 — Supplementary Figure S1. [file 41598_2023_41495_MOESM1_ESM.pdf]

A

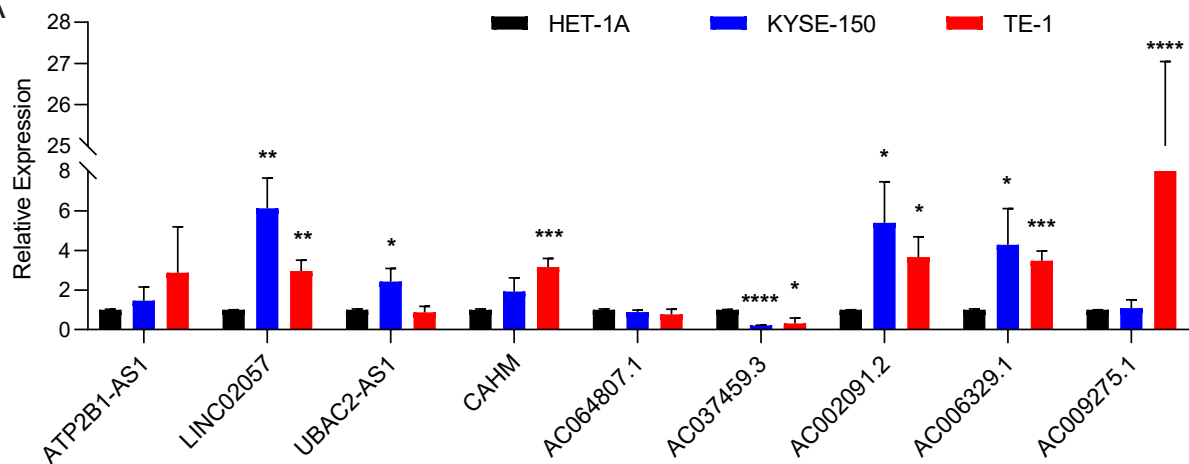

B

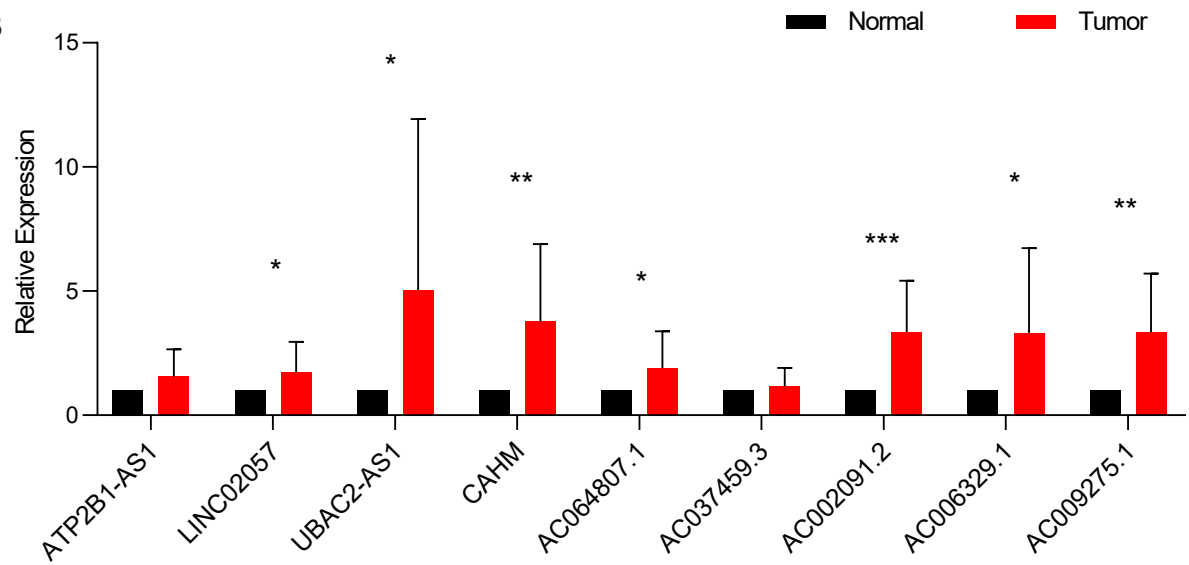

Supplement: Supplementary file 3 — Supplementary Figure S3. [file 41598_2023_41495_MOESM3_ESM.pdf]

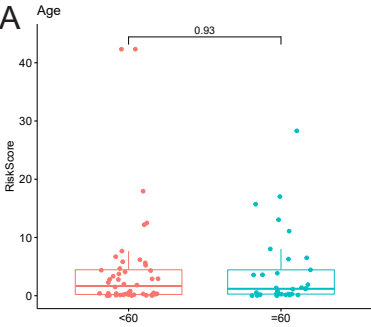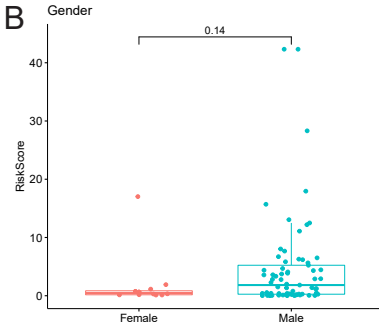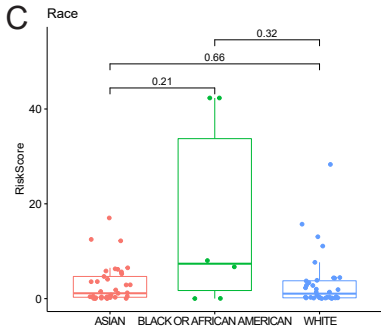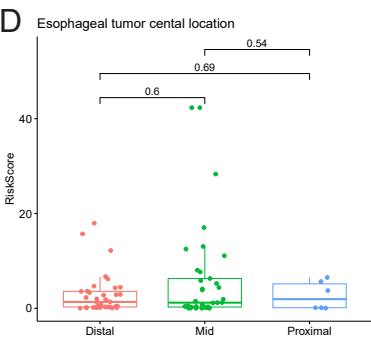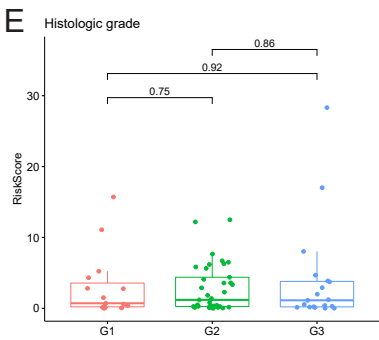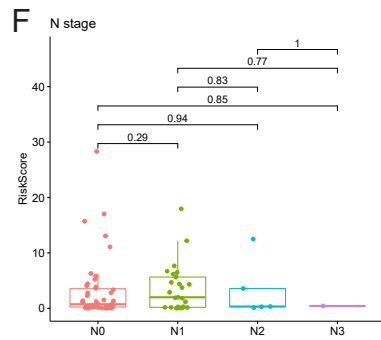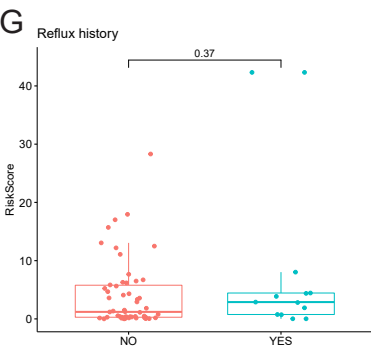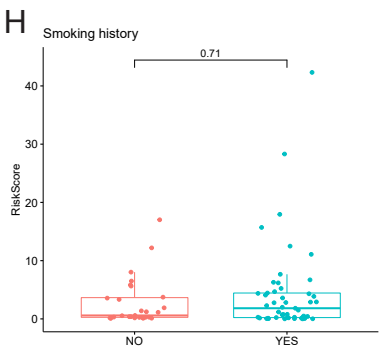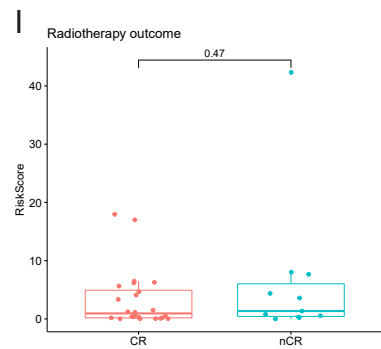

Supplement: Supplementary file 4 — Supplementary Figure S4. [file 41598_2023_41495_MOESM4_ESM.pdf]

A

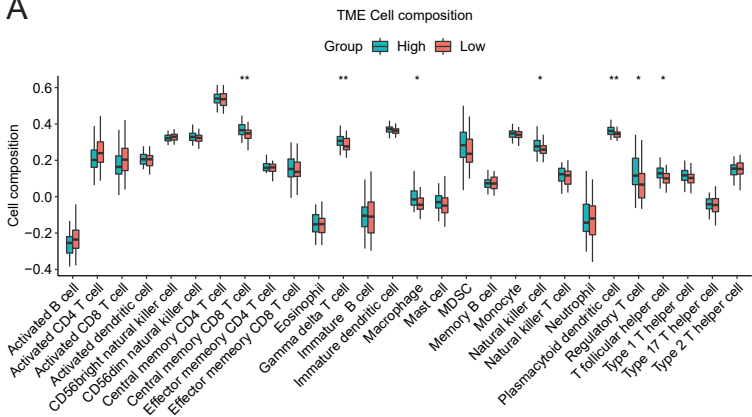

B

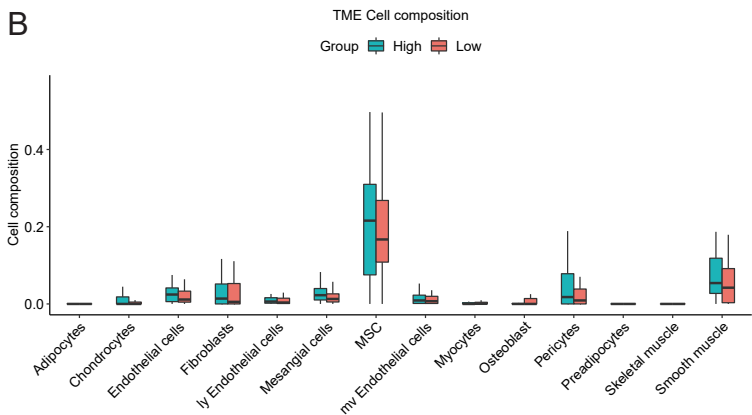

Supplement: Supplementary file 5 — Supplementary Figure S5. [file 41598_2023_41495_MOESM5_ESM.pdf]

B

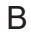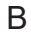

Supplement: Supplementary file 6 — Supplementary Figure S6. [file 41598_2023_41495_MOESM6_ESM.pdf]

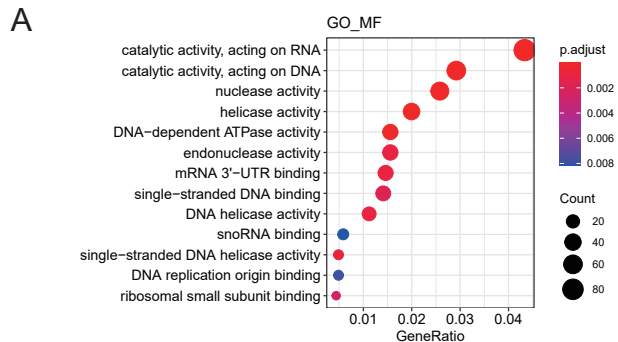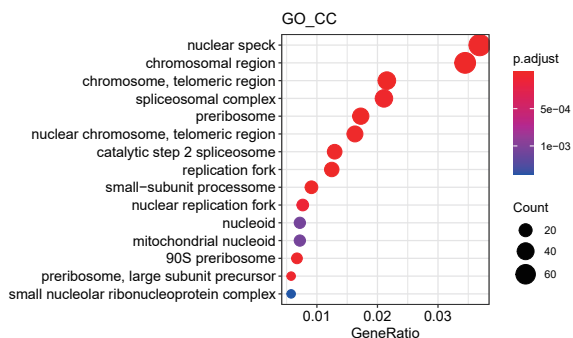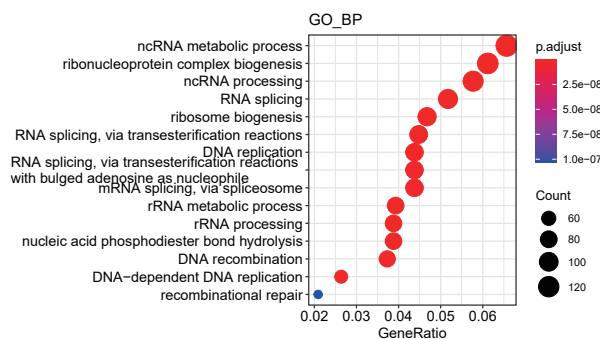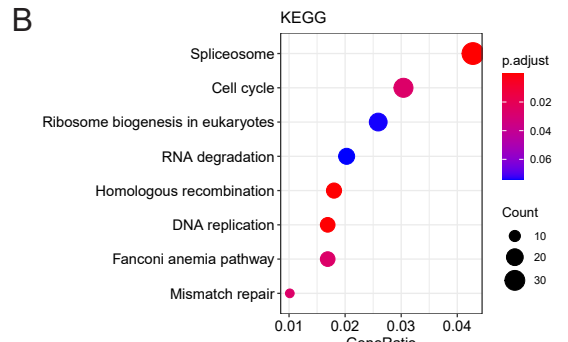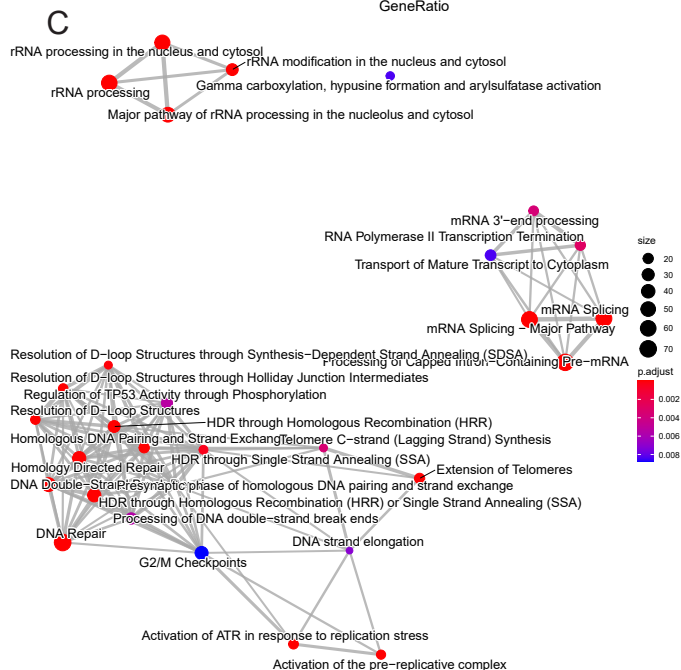

Supplement: Supplementary file 7 — Supplementary Figure S7. [file 41598_2023_41495_MOESM7_ESM.pdf]

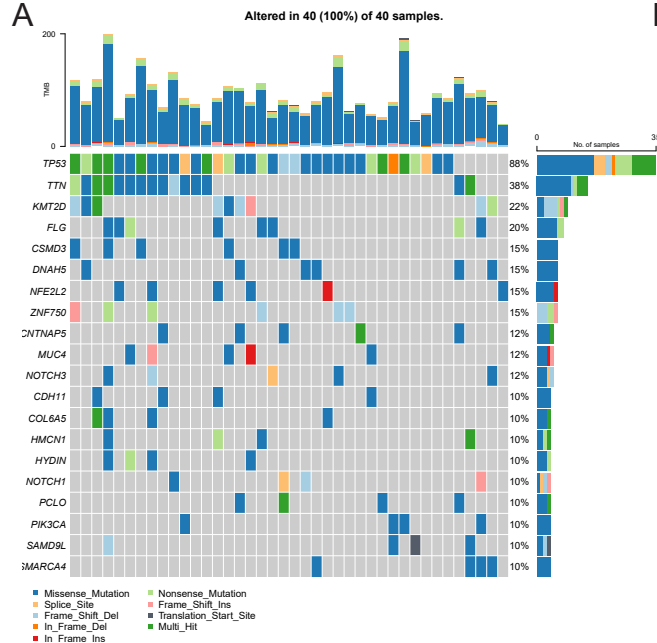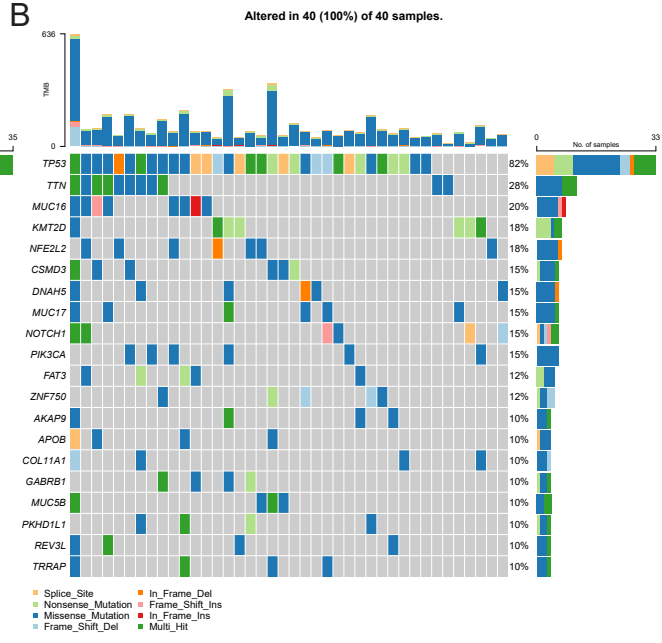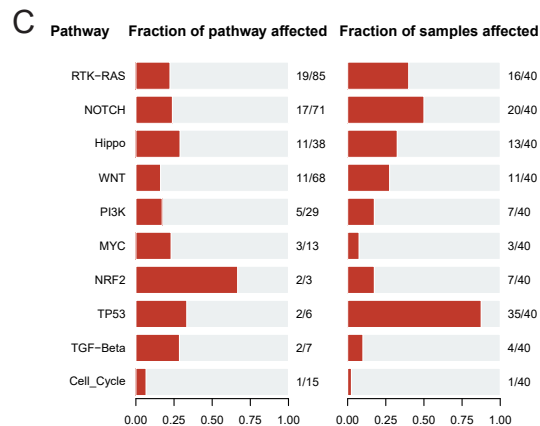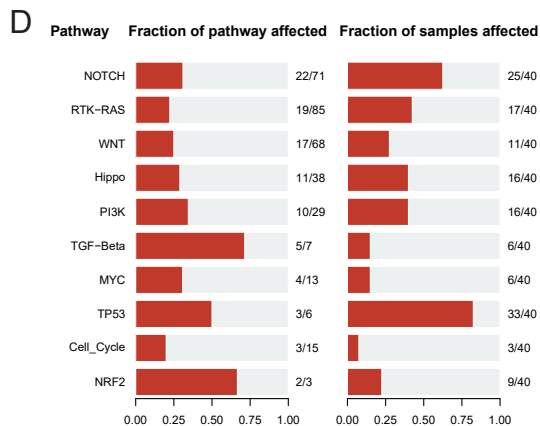

Supplement: Supplementary file 8 — Supplementary Figure S8. [file 41598_2023_41495_MOESM8_ESM.pdf]
