# Supplementary material for: Identification of m5C-related lncRNAs signature to predict prognosis and therapeutic responses in esophageal squamous cell carcinoma patients
Source: Sci Rep. 2023 Sep 4;13:14499. doi: 10.1038/s41598-023-41495-6 (PMC10477299; doi:10.1038/s41598-023-41495-6)
Supplement: Supplementary file 2 — Supplementary Figure S2. [file 41598_2023_41495_MOESM2_ESM.pdf]

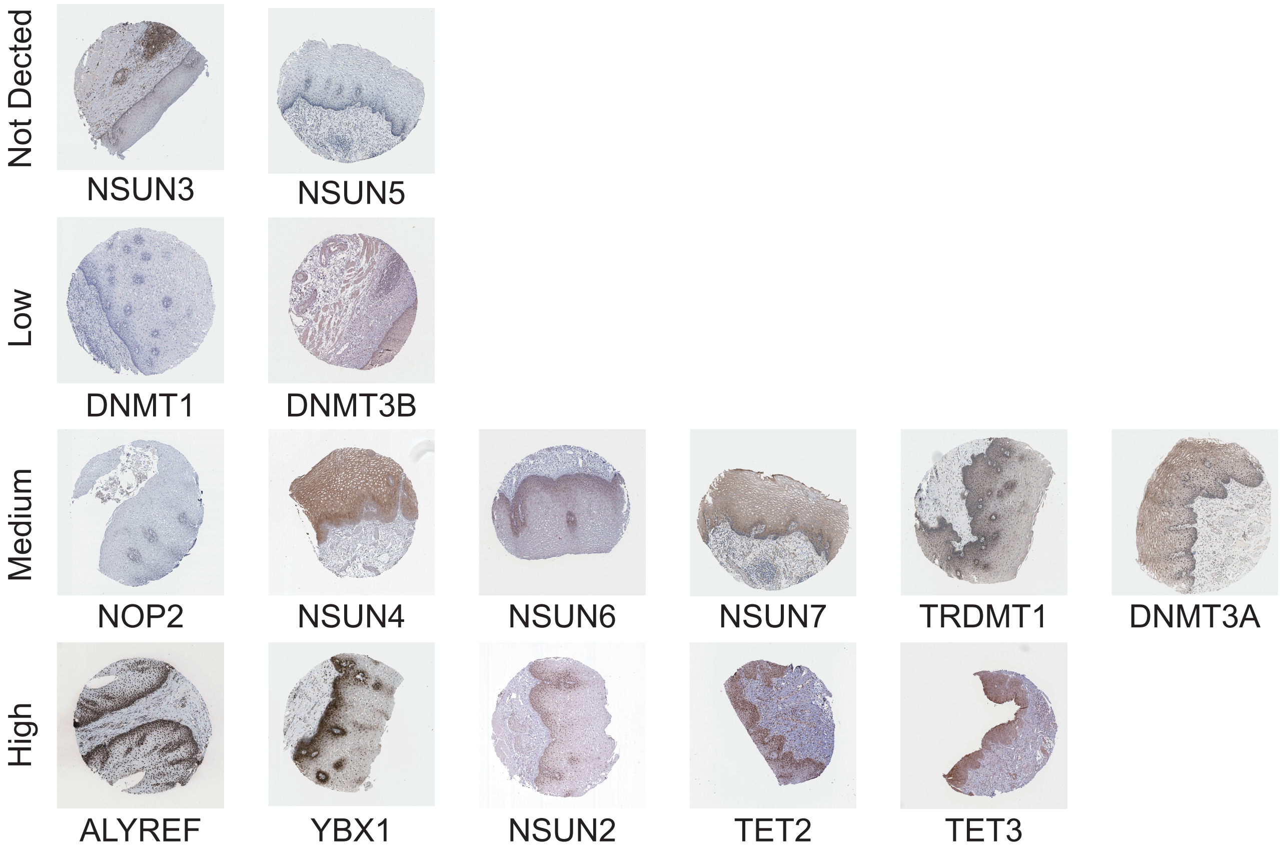
Not Dected
NSUN3
NSUN5
Low
DNMT1
DNMT3B
Medium
NOP2
NSUN4
NSUN6
NSUN7
TRDMT1
DNMT3A
High
ALYREF
YBX1
NSUN2
TET2
TET3
