# Supplementary material for: Identification of m5C-related lncRNAs signature to predict prognosis and therapeutic responses in esophageal squamous cell carcinoma patients
Source: Sci Rep. 2023 Sep 4;13:14499. doi: 10.1038/s41598-023-41495-6 (PMC10477299; doi:10.1038/s41598-023-41495-6)
Supplement: Supplementary file 9 — Supplementary Information. [file 41598_2023_41495_MOESM9_ESM.pdf]

Supplementary Material

Supplementary Figures

Figure S1 Schematic diagram of the study design.

Figure S2 Immunohistochemical analysis of the protein expression of the m^5C regulators in the HPA database.

Figure S3 The lncRNA expression in ESCC cell lines and patients. **(A)** The expression level of m^5C-related lncRNAs in normal esophageal cell line HET-1A and ESCC cell lines KYSE150 and TE-1. **(B)** The expression level of m^5C-related lncRNAs in adjacent normal tissues and ESCC tissues. *$P < 0.05$, **$P < 0.01$, ***$P < 0.001$, and ****$P < 0.0001$.

Figure S4 The discrepancy in risk scores between different subgroups: Age **(A)**, Gender **(B)**, Race **(C)**, Esophageal tumor central location **(D)**, Histologic grade **(E)**, N stage **(F)**, Reflux history **(G)**, Smoking history **(H)**, and Radiotherapy outcome **(I)**.

Figure S5 Comparison of immune and stromal cells infiltration in different risk groups. **(A)** Boxplots showing the infiltration of the immune cells based on the ssGSEA algorithm in different risk groups. **(B)** Comparisons of stromal cells infiltration based on xCell algorithm in different risk groups. *$P < 0.05$ and **$P < 0.01$.

Figure S6 Investigation of immune status in different risk groups. **(A)** Correlation heatmap showing the Pearson correlation among immune and stromal cells. **(B)** Heatmap showing the expression level of immunoinhibitors and immunostimulators in different risk groups.

Figure S7 Functional enrichment analysis of the different risk groups. GO enrichment **(A)** and KEGG pathway **(B)** analyses were performed with the differentially expressed genes. **(C)** Reactome pathway enrichment map for differentially expressed genes. Node color: significance of the enrichment pathway; Node size: the number of genes in the enrichment pathway.

Figure S8 Genomic alterations between different risk groups. Waterfall plots illustrating the top 20 most frequently mutant genes in low-risk **(A)** and high-risk **(B)** groups. Oncogenic pathways alterations fraction in low-risk **(C)** and high-risk **(D)** groups.

Supplementary Table

Table S1 The clinicopathological parameters of TCGA-ESCC and GSE53622 cohorts

		**TCGA-ESCC**	**GSE53622**	***P***
n		80	60	
Age(mean (SD))		58.42 (10.52)	59.98 (9.26)	0.3634
Gender(%)				0.4999
	Male	68 (85.0)	48 (80.0)	
	Female	12 (15.0)	12 (20.0)	
T stage (%)				0.0065
	T1	8 (10.0)	4 (6.7)	
	T2	27 (33.8)	7 (11.7)	

	T3	40 (50.0)	48 (80.0)	
	T4	3 (3.8)	1 (1.7)	
	UNKNOW	2 (2.5)	0 (0）	
N stage (%)				0.1880
	N0	46 (57.5)	29 (48.3)	
	N1	25 (31.2)	20 (33.3)	
	N2	5 (6.2)	9 (15.0)	
	N3	1 (1.2)	2 (3.3)	
	UNKNOW	3 (3.8)	0 (0）	
Stage (%)				0.1163
	Stage I	7 (8.8)	4 (6.7)	
	Stage II	47 (58.8)	30 (50.0)	
	Stage III	21 (26.2)	26 (43.3)	
	Stage IV	3 (3.8)	0 (0）	
	UNKNOW	2 (2.5)	0 (0）	
Tumor cental location (%)				0.7805
	Distal	37 (46.2)	26 (43.3)	
	Mid	36 (45.0)	28 (46.7)	
	Proximal	6 (7.5)	6 (10.0)	
	UNKNOW	1 (1.2)	0 (0）	
Histologic grade (%)				0.0451
	G1	15 (18.8)	9 (15.0)	
	G2	37 (46.2)	34 (56.7)	
	G3	19 (23.8)	17 (28.3)	
	UNKNOW	9 (11.2)	0 (0）	

Table S2 The primer sequences involved in this study

**Primer**	**Forward 3'-5'**	**Reverse 3'-5'**
SRP9	TCCCGGACGTAGGTAGTTTG	CTTCATAGGGTCAGCGAGGT
UBAC2−AS1	TGAAACGATGGCGGTCAGAA	TCAGGTCCTCAGGATGCAGA

LINC02057	AAGGCAGGCTTCGGAAATGA	CCACCTCCGGCAAATCACTT
AC002091.2	ATGTATGAGGGCTCCTGGGT	TGAATACTCTAGCCGCGCAG
AC006329.1	CGAGTTATGCAAACGAGGCG	TCTTCGCTTCCCCAACCTTC
ATP2B1−AS1	GCTCTGACGTCTGTGTTTCCA	AAGTGAAGGGCGTCCCACT
CAHM	AGGGGAGCGTCAGTCGTGCT	TGCGGCTTCATTCCCTCACGG
AC064807.1	ACAGAAAAGAGGGAAGCGGG	TCTCTGAACGCCTTTGGACC
AC037459.3	CAGCTCCTGGTCACGTTCAT	TTCGTTAATTGCGCGCGTAG
AC009275.1	CACAGGTGCAGACGTGTTTG	CCCAATTTCAACCCGCGTTT
GAPDH	GAAGAGAGAGACCCTCACGCTG	ACTGTGAGGAGGGGAGATTCAGT

Table S3 The PPI network of m5C regulators based on STRING database

**node1**	**node2**	**coexpression**	**experimentally_determined_interaction**	**combined_score**
ALYREF	NSUN2	0.082	0.101	0.783
ALYREF	YBX1	0.204	0.489	0.694
ALYREF	NOP2	0.102	0.3	0.569
DNMT1	DNMT3A	0.049	0.696	0.998
DNMT1	DNMT3B	0.101	0.876	0.998
DNMT1	TET1	0	0	0.837
DNMT1	TET3	0	0	0.856
DNMT1	TET2	0	0	0.896
DNMT3A	TET3	0.062	0	0.781

DNMT 3A	TRDMT1	0	0.068	0.813
DNMT 3A	TET1	0.066	0	0.828
DNMT 3A	TET2	0.062	0.27	0.915
DNMT 3A	DNMT3B	0.064	0.818	0.982
DNMT 3A	DNMT1	0.049	0.696	0.998
DNMT 3B	DNMT3A	0.064	0.818	0.982
DNMT 3B	TET3	0	0	0.769
DNMT 3B	TET1	0.213	0	0.825
DNMT 3B	TRDMT1	0	0.068	0.839
DNMT 3B	TET2	0	0	0.859
DNMT 3B	DNMT1	0.101	0.876	0.998
NOP2	NSUN2	0.823	0	0.887
NOP2	NSUN5	0.235	0	0.505
NOP2	NSUN3	0.464	0	0.651
NOP2	TRDMT1	0.064	0	0.798
NOP2	NSUN7	0.159	0	0.789
NOP2	ALYREF	0.102	0.3	0.569
NOP2	NSUN4	0.464	0	0.658
NSUN2	YBX1	0.09	0.27	0.535
NSUN2	NSUN6	0.464	0	0.645
NSUN2	ALYREF	0.082	0.101	0.783
NSUN2	NSUN7	0.124	0	0.823
NSUN2	NOP2	0.823	0	0.887
NSUN2	TRDMT1	0.066	0	0.93
NSUN3	NSUN5	0.124	0	0.864
NSUN3	NOP2	0.464	0	0.651
NSUN3	NSUN6	0.464	0	0.689
NSUN3	TRDMT1	0.066	0	0.742
NSUN3	NSUN7	0.124	0	0.821
NSUN4	NSUN5	0.131	0	0.811
NSUN4	NSUN6	0.464	0	0.644
NSUN4	TRDMT1	0.066	0	0.716

NSUN4	NSUN7	0.124	0	0.782
NSUN4	NOP2	0.464	0	0.658
NSUN5	NOP2	0.235	0	0.505
NSUN5	NSUN6	0.159	0	0.52
NSUN5	TRDMT1	0.064	0	0.762
NSUN5	NSUN4	0.131	0	0.811
NSUN5	NSUN3	0.124	0	0.864
NSUN6	NSUN2	0.464	0	0.645
NSUN6	NSUN5	0.159	0	0.52
NSUN6	NSUN3	0.464	0	0.689
NSUN6	NSUN4	0.464	0	0.644
NSUN6	TRDMT1	0.064	0	0.789
NSUN6	NSUN7	0.159	0	0.808
NSUN7	NSUN2	0.124	0	0.823
NSUN7	NSUN3	0.124	0	0.821
NSUN7	NSUN6	0.159	0	0.808
NSUN7	TRDMT1	0.064	0	0.666
NSUN7	NSUN4	0.124	0	0.782
NSUN7	NOP2	0.159	0	0.789
TET1	DNMT3A	0.066	0	0.828
TET1	DNMT3B	0.213	0	0.825
TET1	DNMT1	0	0	0.837
TET1	TRDMT1	0	0	0.515
TET1	TET3	0	0	0.707
TET1	TET2	0.063	0	0.724
TET2	DNMT3A	0.062	0.27	0.915
TET2	DNMT3B	0	0	0.859
TET2	DNMT1	0	0	0.896
TET2	TET1	0.063	0	0.724
TET2	TET3	0.063	0.379	0.802
TET3	DNMT3A	0.062	0	0.781
TET3	DNMT3B	0	0	0.769
TET3	DNMT1	0	0	0.856
TET3	TET1	0	0	0.707
TET3	TRDMT1	0	0	0.517
TET3	TET2	0.063	0.379	0.802
TRDMT1	NSUN2	0.066	0	0.93
TRDMT1	DNMT3A	0	0.068	0.813
TRDMT1	NSUN5	0.064	0	0.762

TRDMT1	NSUN3	0.066	0	0.742
TRDMT1	DNMT3B	0	0.068	0.839
TRDMT1	TET1	0	0	0.515
TRDMT1	NSUN6	0.064	0	0.789
TRDMT1	TET3	0	0	0.517
TRDMT1	NSUN7	0.064	0	0.666
TRDMT1	NSUN4	0.066	0	0.716
TRDMT1	NOP2	0.064	0	0.798
YBX1	NSUN2	0.09	0.27	0.535
YBX1	ALYREF	0.204	0.489	0.694

Table S4 m5C-related lncRNAs with prognosis value

**lncRNA**	**HR**	**HR.95L**	**HR.95H**	**P value**
AP002498.1	2.4	1.051	5.477	0.038
LINC00993	3.189	1.175	8.651	0.023
LINC00624	3.22	1.269	8.172	0.014
SPART-AS1	0.384	0.165	0.892	0.026
HOXA11-AS	2.383	1.043	5.447	0.04
SLC47A1P2	2.439	1.032	5.764	0.042
AL132989.1	2.473	1.025	5.964	0.044
SNHG28	0.43	0.187	0.991	0.047
AC105001.1	3.423	1.4	8.366	0.007
KIF25-AS1	2.641	1.083	6.439	0.033
LINC01006	0.386	0.16	0.929	0.034
AC100861.1	2.321	1.013	5.319	0.047
ATP2B1-AS1	2.398	1.047	5.49	0.038
AC107072.1	3.066	1.143	8.221	0.026
LINC01515	2.684	1.137	6.338	0.024
AC005288.1	2.448	1.069	5.605	0.034
AC109587.1	2.833	1.179	6.808	0.02
AC087318.1	2.904	1.21	6.97	0.017

LINC02057	0.433	0.19	0.985	0.046
EGFEM1P	2.472	1.063	5.748	0.036
AL158166.1	0.384	0.169	0.875	0.023
LINC01486	5.688	1.337	24.194	0.019
GUSBP6	0.337	0.148	0.768	0.01
UBAC2-AS1	2.595	1.11	6.067	0.028
CAHM	3.156	1.256	7.93	0.015
CDKN2B-AS1	2.391	1.047	5.459	0.039
AC064807.1	3.691	1.456	9.354	0.006
AC114546.1	3.285	1.293	8.344	0.012
AC015987.1	3.008	1.182	7.656	0.021
ACBD3-AS1	2.372	1.009	5.575	0.048
AC083843.2	2.6	1.071	6.312	0.035
AL390198.1	0.413	0.181	0.943	0.036
AC037459.3	2.573	1.124	5.891	0.025
AC002091.2	0.344	0.147	0.805	0.014
AL392172.1	2.534	1.11	5.786	0.027
AL353625.1	3.274	1.359	7.887	0.008
UBL7-AS1	3.951	1.511	10.332	0.005
AC016065.1	2.369	1.038	5.407	0.04
AC006329.1	3.189	1.319	7.712	0.01
AL133371.3	0.372	0.163	0.849	0.019
AC009275.1	0.402	0.175	0.924	0.032
